# The metabolism-immune axis in colorectal cancer: remodeling the tumor microenvironment through metabolite signaling

**DOI:** 10.3389/fimmu.2025.1735873

**Published:** 2025-12-19

**Authors:** Shaofan Hu, Hui Heng, Fang Yang, Meng Wang, Guoxiang Liu, Yuancai Xiang, Hongming Miao

**Affiliations:** 1 Center of Smart Laboratory and Molecular Medicine, School of Medicine, Chongqing University, Chongqing, China; 2 College of Bioengineering, Chongqing University, Chongqing, China; 3 Jinfeng Laboratory, Chongqing, China; 4 Department of Pathophysiology, College of High Altitude Military Medicine, Third Military Medical University (Army Medical University), Chongqing, China; 5 Department of Physiology, School of Basic Medical Sciences, Southwest Medical University, Luzhou, Sichuan, China; 6 Key Laboratory of Extreme Environmental Medicine, Ministry of Education of China, Third Military Medical University (Army Medical University), Chongqing, China; 7 Department of Biochemistry and Molecular Biology, School of Basic Medical Sciences, Southwest Medical University, Luzhou, Sichuan, China

**Keywords:** colorectal cancer (CRC), metabolic reprogramming, tumor microenvironment, immune evasion, gut microbiota, metabolism-targeted therapy

## Abstract

Metabolic reprogramming is a defining hallmark of tumors, and plays a pivotal role in sustaining malignant growth by rewiring core bioenergetic and biosynthetic pathways. Beyond supporting tumor cell proliferation, survival, and metastasis, it profoundly shapes the tumor microenvironment through nutrient competition, accumulation of immunosuppressive metabolites, and modulation of immune cell function, thereby facilitating immune evasion and therapy resistance. This review comprehensively elaborates on metabolic reprogramming in colorectal cancer, covering key alterations in glucose metabolism (Warburg effect), tricarboxylic acid cycle remodeling, lipid biosynthesis/oxidation, cholesterol metabolism, and amino acid (glutamine, methionine, tryptophan, arginine) metabolism. It further dissects how these metabolic shifts impact the tumor microenvironment in colorectal cancer, including their effects on effector immune cells (CD8^+^ T cells, NK cells), immunosuppressive populations (Tregs, MDSCs, M2-TAMs), and antigen-presenting cells. Additionally, this review highlights the role of the gut microbiota and their metabolites (e.g., SCFAs, secondary bile acids and indoles) in remodeling the immune microenvironment via metabolic crosstalk. Overall, this work provides a comprehensive understanding of CRC metabolic reprogramming and its microenvironmental impacts, offering critical insights to guide the development of novel metabolism-targeted therapeutic strategies for CRC.

## 1 Background

Colorectal cancer (CRC) is a highly prevalent and aggressive malignancy of global significance, ranking among the leading causes of cancer-related morbidity and mortality worldwide. Its incidence varies substantially across geographic regions, with notable disparities between developed and developing nations. A striking epidemiological trend in recent years is the shifting age distribution of onset, marked by a gradual rise in the proportion of young patients alongside a concurrent decline in elderly cases (1).

The pathogenesis of CRC arises from a complex interplay of multiple factors. Environmental and lifestyle influences exert profound effects: unhealthy dietary patterns (such as excessive red meat consumption and inadequate dietary fiber intake) and detrimental behaviors (including physical inactivity, smoking, and alcohol abuse) significantly increase disease risk (2). Metabolic syndrome-associated conditions, particularly obesity and type 2 diabetes, are also strongly linked to CRC development (3). Concurrently, gut microbiota dysbiosis plays a critical role in driving disease initiation and progression. Additionally, genetic predisposition is a key consideration, with a subset of cases attributed to well-defined hereditary syndromes, and a positive family history representing a major risk factor.

Metabolic reprogramming stands as a core hallmark of CRC. To sustain rapid proliferation and survival, cancer cells rewire core metabolic pathways, including enhanced aerobic glycolysis, increased glutamine dependency, and altered lipid metabolism and amino acid utilization. These metabolic adaptations not only supply energy and biosynthetic precursors for tumor cells but also profoundly shape the tumor microenvironment (TME). Within the TME, cancer cells compete with immune cells for nutrients and modulate metabolite concentrations, thereby remodeling the immune landscape to promote immune evasion and therapeutic resistance. As metabolism constitutes a primary driver of tumor growth, understanding metabolic reprogramming in CRC cells and the TME can identify direct therapeutic targets for disease prevention and treatment, while precision interventions based on this knowledge can augment the efficacy of other therapeutic modalities.

This review explores the specific mechanisms underlying metabolic reprogramming in CRC, elucidates how these metabolic alterations influence the TME and immune cell function, and discusses future directions for metabolism-targeted therapies. Its aim is to provide novel insights and strategies to advance CRC treatment and improve patient outcomes.

## 2 Overview of colorectal cancer

Colorectal cancer, the third most prevalent malignant tumor worldwide and the second leading cause of cancer-related mortality, arises from a complex interplay of genetic mutations, environmental exposures, metabolic abnormalities, and tumor microenvironment remodeling. Epidemiological data reveal that in 2022, there were more than 1.9 million new global cases and approximately 934,000 deaths attributed to CRC (4), accounting for nearly one-tenth of the total cancer cases and fatalities worldwide.

Notably, the incidence of CRC exhibits substantial geographical disparities, with rates in developed countries exceeding four times those in developing nations. This discrepancy may be attributed to factors such as increased consumption of animal-derived foods, sedentary lifestyles associated with elevated obesity rates, and variations in screening accessibility. A striking trend is the declining overall number of elderly patients alongside a sharp rise in the proportion of young and middle-aged patients, reflecting a rapid shift toward younger-onset CRC. As indicated in the American Cancer Society’s 2023 report, the overall mortality rate of CRC decreased by 2% annually between 2011 and 2020; however, the proportion of young and middle-aged patients surged significantly. Specifically, among individuals under 55 years of age, the share of newly diagnosed cases doubled from 11% in 1995 to 20% in 2019 (5), whereas the incidence rate among those aged 50 years and above has steadily declined by approximately 3%–5% annually since 2000.

## 3 Etiological mechanisms of colorectal cancer

Colorectal cancer develops from the intricate interplay of environmental and genetic factors (6, 7). A systematic review by the World Cancer Research Fund (WCRF) indicated that excessive consumption of red meat—particularly processed meat—increases CRC risk by 17–19%, whereas each additional 10 g/day of dietary fiber intake is associated with an approximately 10% risk reduction. Epidemiological evidence specific to women shows that daily consumption of ≥2 servings of sugar-sweetened beverages elevates CRC risk by 118%, whereas studies on young-onset CRC reveal inverse correlations between disease risk and intake of fruits, vegetables, dietary fiber, β-carotene, and vitamins C/E (8, 9).

Lifestyle factors significantly affect CRC susceptibility: daily alcohol intake exceeding 50 g increases the risk by over more than 41% (2), a 20 pack-year smoking history in individuals aged 30–39 raises the risk by 341% (10), and prolonged sedentary behavior (≥14 hours/week) is linked to a 68% increased risk of early-onset CRC and a 162% increased risk of rectal cancer in women (11). Conversely, high levels of physical activity correlate with reduced risks of colon cancer, rectal cancer, and overall CRC (12).

Among metabolic syndrome-related factors, a body mass index (BMI) ≥30 kg/m² increases CRC risk by 93% in young women (13); type 2 diabetes elevates CRC incidence by over 64% in elderly patients (>55 years) (3); and chronic inflammatory bowel disease is associated with increased sporadic CRC risk (1). Additionally, gut microbiota dysbiosis contributes to CRC pathogenesis: high abundance of *Fusobacterium nucleatum* is linked to a 58% increase in mortality (14), while enterotoxigenic *Escherichia coli* (harboring the polyketide synthase genomic island) can promote carcinogenesis by inducing DNA double-strand breaks (15).

At the genetic level, approximately 5–7% of CRC cases are linked to well-defined hereditary syndromes (16). These include Lynch syndrome (hereditary nonpolyposis colorectal cancer, HNPCC), caused by germline mutations in mismatch repair genes (e.g., MLH1, MSH2), accounting for 2–3% of all CRC cases and characterized by high microsatellite instability (MSI-H); familial adenomatous polyposis (FAP), where APC mutations lead to the development of hundreds to thousands of adenomas with a nearly 100% risk of malignant transformation by age 40; and other polyposis syndromes (e.g., MUTYH-associated polyposis, Peutz-Jeghers syndrome), which drive carcinogenesis via distinct molecular pathways and collectively account for ~1% of CRC cases. Approximately 10–20% of patients have a positive family history, with first-degree relatives exhibiting a 2–4-fold increased risk. Genome-wide association studies have identified over 40 common single nucleotide polymorphisms (SNPs) associated with CRC risk, with individual SNP odds ratios (ORs) typically ranging from 1.1 to 1.3.

CRC pathogenesis involves two distinct precursor pathways: the adenoma-carcinoma pathway, characterized by chromosomal instability (CIN) and accounting for 60–70% of cases (17), which typically initiates with APC mutations followed by RAS activation or TP53 functional loss; and the serrated neoplasia pathway, associated with KRAS and BRAF mutations and epigenetic instability, leading to both microsatellite-stable (MSS) and microsatellite instability (MSI) CRC. Among them MSI-CRC frequently arises from MLH1 promoter hypermethylation. Approximately 15%-18% of patients with stage II colorectal cancer (CRC), 9%-10% with stage III CRC, and 4%-5% with metastatic CRC (mCRC) exhibit phenotypes of microsatellite instability and/or mismatch repair deficiency (dMMR, caused by functional defects in MMR-related proteins) (18).

Sporadic CRC exhibits marked molecular heterogeneity. Nearly all CIN tumors display activated Wnt signaling, with 80% harboring APC inactivation mutations (17) and 5% carrying CTNNB1 activating mutations, resulting in abnormal β-catenin accumulation (19). TP53 mutation/inactivation or deletion occurs in 60% of CIN tumors (17), with p53 functional loss directly driving CIN and facilitating genomic instability. Activating mutations in KRAS, NRAS, or HRAS (at codons 12 or 13) are present in ~50% of CRC cases. The RAS effector BRAF is mutated in 10–15% of early-stage CRC cases (20) and ~5% of stage IV CRC cases, with mutations predominantly occurring at hotspot codon (V600E) and being mutually exclusive with RAS mutations (21). In CRC, the tumor suppressor gene SMAD4 is mutated in 12% of patients with metastatic or unresectable disease and represents one of the most commonly mutated genes in metastases compared with those in primary tumors (22).

## 4 Metabolic reprogramming in colorectal cancer core pathway alterations

Colorectal cancer is characterized by profound metabolic rewiring that sustains malignant progression through dysregulation of core bioenergetic and biosynthetic pathways. Central to this reprogramming is enhanced aerobic glycolysis (Warburg effect), wherein cancer cells preferentially convert glucose to lactate despite oxygen availability, providing rapid ATP generation and glycolytic intermediates for nucleotide/lipid synthesis. Concurrently, CRC cells exhibit glutamine addiction—diverting this abundant amino acid toward anaplerotic replenishment of the tricarboxylic acid (TCA) cycle, non-essential amino acid production, and redox homeostasis. Lipid metabolism is extensively altered, marked by hyperactivation of *de novo* lipogenesis and increased exogenous fatty acid uptake, supporting membrane biogenesis, lipid droplet storage, and signaling molecule synthesis. The TCA cycle undergoes context-dependent remodeling, with mutations (e.g., KRAS, TP53) or hypoxia driving accumulation of oncometabolites (succinate, fumarate) and aberrant flux. Amino acid metabolism, particularly methionine cycling, tryptophan-kynurenine shunting, and arginine-polyamine conversion, is co-opted to fuel epigenetic dysregulation, redox balance, and nucleotide pools. These multidimensional metabolic alterations not only meet the bioenergetic and biosynthetic requirements of rapidly proliferating tumor cells but also remodel the tumor microenvironment through metabolic crosstalk, thereby promoting immune evasion and therapy resistance.

### 4.1 The Warburg effect: aerobic glycolysis in colorectal cancer

Colorectal cancer cells exhibit a universal enhancement in glycolysis (Warburg effect), preferentially utilizing glycolysis over oxidative phosphorylation for energy production even under oxygen-replete conditions. This metabolic reprogramming sustains rapid proliferation by supplying energy and biosynthetic precursors while promoting immune evasion and therapy resistance through microenvironment remodeling. Normal colorectal epithelial cells primarily rely on mitochondrial oxidative phosphorylation (OXPHOS) for ATP generation, while transformed CRC cells aberrantly upregulate glycolysis, converting large amounts of glucose to lactate regardless of oxygen availability. This metabolic shift is driven by aberrant expression of key metabolic enzymes and dysregulation of hierarchical regulatory networks.

Constitutively activated Wnt signaling represents a central driver of CRC development: Wnt pathway activation induces the transcription of downstream targets such as c-Myc (23), which directly upregulates GLUT1 and hexokinase 2 (HK2) while repressing the transcription of the mitochondrial pyruvate carrier (MPC), thereby diverting pyruvate toward lactate production rather than the TCA cycle. In colorectal cancer, MPC expression is significantly downregulated, with low expression correlating with poor prognosis (24). MPC overexpression can fully abrogate the proliferation of intestinal stem cells deficient in Apc or Notch tumor suppressors, whereas MPC knockout accelerates malignant tumor proliferation in the AOM/DSS model—concurrently with activation of the Wnt/β-catenin signaling pathway. In addition, in colorectal cancer the long noncoding RNA LINC01764 promotes the malignant proliferation of colorectal cancer and resistance to 5-FU by activating c-Myc to upregulate glucose and glutamine metabolic pathways (25). Conversely the tumor suppressor p53 inhibits glycolysis by downregulating GLUT1, GLUT3, and GLUT4 to limit glucose uptake (26), while directly or indirectly modulating glycolytic enzymes including HK2, G6PD, PFKFB3/4, PGAM1, and PHGDH (27). Besides, recent studies show that METTL14, transcriptionally activated by wild-type p53 in CRC cells, suppresses SLC2A3 and PGAM1 expression to attenuate aerobic glycolysis (28). Loss or downregulation of p53 in CRC alleviates this inhibitory constraint, exacerbating glycolytic flux.

Oncogenic mutations in KRAS are critical drivers of colorectal cancer, studies have reported that the upregulation of GLUT1 expression and increased glucose uptake primarily depend on KRAS and BRAF mutations in CRC cells. This metabolic reprogramming confers a distinct survival advantage, enabling CRC cells harboring KRAS or BRAF mutations to achieve long-term survival under low-glucose culture conditions (29). Enhanced glycolysis in CRC is also linked to the activation of HIF1A, PI3K/AKT, and mTOR pathways (30). In microenvironments hypoxia stabilizes HIF1α through the inhibition of prolyl hydroxylases (PHDs), thereby driving upregulation of glycolytic genes (e.g., GLUT, HK and PGK) and PDK1-mediated suppression of pyruvate dehydrogenase (PDH) (31). In addition, as a target of β-catenin, HIF-1α can cooperate with nuclear β-catenin to regulate transcriptional activity (32). In CRC mitochondrial dysfunction triggers metabolic reprogramming from OXPHOS to glycolysis and the pentose phosphate pathway (PPP), via reactive oxygen species (ROS) and non-canonical Wnt/β-catenin-mediated HIF-1α upregulation, irrespective of oxygen levels (33). The PI3K/AKT signaling pathway plays a crucial role in tumor development and progression, and its aberrant activation is closely associated with proliferation, survival, metastasis, and treatment resistance in various cancers (34). The PI3K/Akt signaling axis is significantly activated in colorectal cancer (23), and enhanced PI3K/Akt signaling not only directly activates GLUT1/4, HK2, and PFKFB3/4 but also coordinates glucose metabolism, nucleotide metabolism, and amino acid synthesis via activation of the mTOR pathway (35).

Notably, dynamic conformational changes in the pyruvate kinase M2 isoform (PKM2) function as a molecular switch governing the Warburg effect: its low-activity dimeric form promotes the accumulation of glycolytic intermediates (e.g., fructose-6-phosphate, 3-phosphoglycerate) to fuel PPP-derived ribose-5-phosphate for nucleotide synthesis and NADPH production, whereas tyrosine phosphorylation modulates its tetrameric active form, enabling metabolic flux partitioning to meet biosynthetic demands (36). In addition to its canonical glycolytic role, nuclear-translocated PKM2 acts as a protein kinase and transcriptional coactivator for HIF1A, NFκB, and β-catenin targets, transactivating genes involved in metabolism and proliferation (36). In CRC, DDX39B enhances PKM2 stabilization and nuclear translocation independently of ERK1/2-mediated phosphorylation, thereby driving the expression of oncogenes and glycolytic genes to accelerate tumor progression (37). Thus, multiple pathways in colorectal cancer promote the enhancement of glycolysis. These findings collectively demonstrate that glycolytic enhancement in colorectal cancer is a tightly regulated metabolic adaptation driven by key oncogenic pathways (Wnt, KRAS, PI3K/AKT/mTOR), tumor suppressor dysfunction (p53) which together support malignancy by prioritizing glycolysis for energy, biosynthesis, and microenvironmental adaptation.

### 4.2 Tricarboxylic acid cycle: metabolic flux and regulatory roles

The TCA cycle—also termed the citric acid cycle or Krebs cycle—functions as the central hub for the oxidative metabolism of glucose and other fuel molecules within mitochondria (39). The TCA cycle begins with acetyl-CoA, derived from glycolysis, fatty acid β-oxidation, or amino acid catabolism (44). The TCA cycle generates high-energy electrons carried by NADH and FADH_2_, which are shuttled to the electron transport chain (ETC). Here, proton gradient-driven OXPHOS produces numerous ATP, underscoring the tight coupling between the TCA cycle and mitochondrial respiration (45). Otto Warburg’s seminal observation, that cancers preferentially ferment glucose to lactate (aerobic glycolysis) even under oxygen-replete conditions, initially suggested mitochondrial respiratory defects as the metabolic basis of malignancy (46, 47). For decades, it has been widely believed that cancer cells primarily rely on aerobic glycolysis while suppressing mitochondrial respiration. However, recent evidence suggests that not all tumors exhibit the characteristic metabolic phenotype of aerobic glycolysis, as several types of cancers maintain functional mitochondria, including intact respiratory processes. Moreover, some tumors even demonstrate elevated levels of OXPHOS (48). Recent studies have shown that primary Kras-mutant tumors (including colorectal cancer) exhibit suppressed TCA cycle flux compared to healthy tissues, however, metastatic lesions often exhibit enhanced flux (49). Nevertheless, cancer cells typically inhabit a harsh metabolic microenvironment marked by oxygen and nutrient scarcity, along with intense competition with other cell types. On the one hand, insufficient oxygen supply may inhibit TCA flux (50, 51), on the other hand, the loss of intrinsic functions in some tumors reduces ATP consumption, prompting inhibition of the TCA cycle to prevent mitochondria from overproducing ATP and citrate (49, 52).

The shift from TCA cycle entry to lactate production is linked to the inhibition of pyruvate dehydrogenase (PDH) by pyruvate dehydrogenase kinase 1 (PDK1). PDH inhibition disconnects the TCA cycle from glycolysis, causing marked reductions in TCA flux and ATP generation. In addition to being regulated by HIF1α, PDK1 can be activated by two kinases, Akt and PGK1, which are translocated to mitochondria to exert their protein kinase activity (52). PDK1 is overexpressed in many cancers, in colorectal cancer its aberrant upregulation is associated with poor prognosis and increased liver metastasis (53). In KRAS-mutant CRC, vitamin C suppresses PDK1 expression by inhibiting HIF-1α stability and transcriptional activity. Accordingly, reduced PDK1 activity activates PDH, increasing TCA cycle flux and promoting colorectal cancer cell apoptosis (54). Phosphoglycerate kinase 1 (PGK1), the first ATP-generating enzyme in glycolysis, catalyzes the conversion of 1,3-bisphosphoglycerate (1,3-BPG) to 3-phosphoglycerate (3-PG) while producing first ATP molecule. In colorectal cancer, however, PGK1 is upregulated, where its O-GlcNAcylation facilitates mitochondrial translocation. Upon mitochondrial entry, PGK1 acts as a protein kinase to phosphorylate PDK1, thereby inhibiting PDH, reducing TCA flux, and enhancing colorectal cancer cell proliferation (55). In colorectal cancer, beyond PGK1 modification, O-GlcNAcylation of c-Myc suppresses the TCA cycle by upregulating the transcription of PDK2. This, in turn, inhibits pyruvate dehydrogenase (PDH)-mediated mitochondrial pyruvate metabolism, reducing mitochondrial pyruvate metabolism and promoting cell proliferation and tumor growth (56).

In addition to energy production, the TCA cycle intermediates, citrate, α-ketoglutarate (α-KG), succinate, and fumarate, play pivotal roles in tumorigenesis (57). Oncogenes and tumor suppressors regulate cycle flux by modulating fuel translocator and enzyme activities (58), with frequent mutations or dysregulation observed in cycle enzymes (aconitase, IDH, fumarate hydratase [FH], SDH and KGDHC) across cancer types (59–61). Recurrent mutations in IDH1/IDH2—prevalent in gliomas (accounting for 80%) (59), acute myeloid leukemia (AML, accounting for 20%) (62, 63), and chondrosarcomas (accounting for 80%) (64, 65) but occurring in <1% of CRC cases (66)—drive neomorphic production of the oncometabolite 2-hydroxyglutarate (2-HG). This metabolite competitively inhibits α-KG-dependent dioxygenases (TETs, JMJD histone demethylases), inducing epigenetic hypermethylation and HIF-1α stabilization to promote chemoresistance and angiogenesis (65). Notably, although the mutation rate of IDH is relatively low, studies have shown that wild-type IDH2 is highly expressed in CRC and can promote CRC progression (67, 68). Conversely, although FH loss is rare in CRC compared to renal cancer (69), its downregulation leads to fumarate accumulation (70), its downregulation leads to fumarate accumulation (70), which can activate oncogenic PI3K/AKT via PTEN succinylation (71) and stimulate HIF1A/Nrf2 pathways to fuel proliferation (72).

Succinate dehydrogenase (SDH) deficiency is another hallmark of metabolic reprogramming in CRC, as a key enzyme linking the TCA cycle and oxidative phosphorylation, SDH activity is critical for maintaining the balance and stability of the electron transport chain (73). Studies have shown that consistent downregulation of SDH expression occurs in both human CRC samples and CRC cell lines (74, 75). Loss of SDH expression is associated with poor patients prognosis, and low SDH expression not only accelerates CRC growth (75) but also promotes CRC invasion and metastasis (74, 76). Inactivation of SDH subunits leads to succinate accumulation, and succinate accumulation in tumor tissues stabilizes HIF-1α by inhibiting prolyl hydroxylases to promote angiogenesis and metastasis (77). Succinate secreted by tumor cells activates the PI3K/HIF-1α and ERK1/2 pathways via the SUCNR-1 receptor, thereby upregulating VEGF expression, inducing angiogenesis, and enhancing cancer cell migration (77). In addition, succinate promotes epithelial-mesenchymal transition (EMT) through STAT3 phosphorylation, increasing the risk of CRC metastasis (78).

Thus, the TCA cycle and its associated metabolic alterations have emerged as critical drivers of colorectal cancer pathogenesis, linking mitochondrial function, oncometabolite accumulation, and malignant progression.

### 4.3 Fatty acid metabolism: synthesis, oxidation, and lipid reprogramming

Fatty acid metabolic reprogramming in colorectal cancer constitutes a fundamental metabolic pillar supporting malignant phenotypes. Through tightly regulated *de novo* synthesis, uptake/storage, modification, and catabolism of fatty acids, it sustains cell proliferation, metastatic dissemination, and therapy resistance. For decades, fatty acid synthesis has been recognized as a key hallmark of tumors. Normal tissues primarily obtain lipids from the bloodstream, with the exception of the liver, adipose tissue, and lactating mammary glands—where high expression of fatty acid synthase (FASN) drives increased *de novo* fatty acid synthesis (79). Even under normoxic conditions, cancer cells synthesize more than 90% of cellular lipids *de novo* to meet biosynthetic demands during rapid proliferation (80, 81).

**Figure 1:**
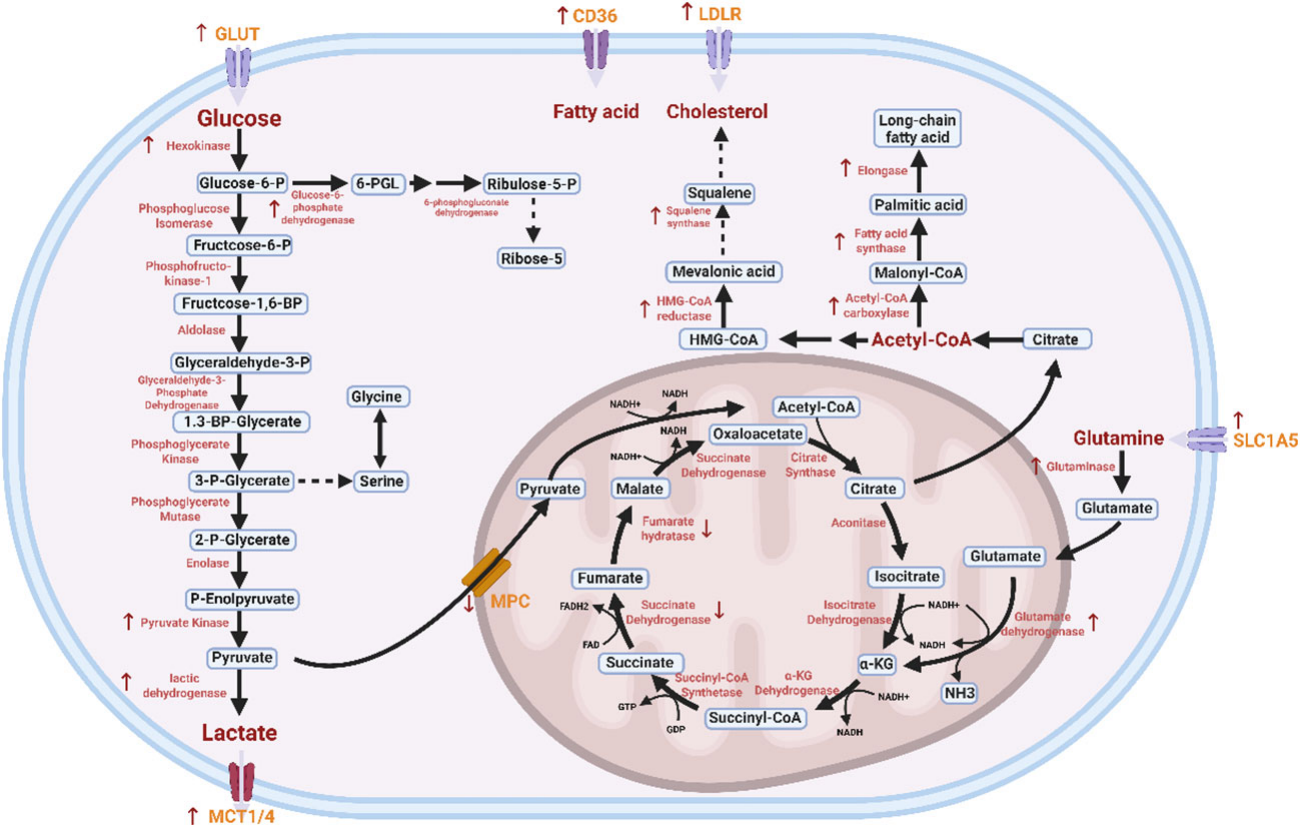
Glucose and lipid metabolic pathways in colorectal cancer cells. Glucose is sequentially converted to pyruvate via a series of enzymatic reactions, catalyzed by hexokinase (HK), phosphoglucose isomerase (PGI), phosphofructokinase-1 (PFK-1), aldolase (ALDO), glyceraldehyde-3-phosphate dehydrogenase (GAPDH), phosphoglycerate kinase (PGK), phosphoglycerate mutase (PGAM), enolase (ENO), and pyruvate kinase (PK). Among these enzymes, HK, PFK-1, and PK act as rate-limiting enzymes. Under hypoxic conditions, pyruvate is further catalyzed by lactate dehydrogenase (LDH) to form lactate (38). Pyruvate can enter mitochondria via transport mediated by the mitochondrial pyruvate carrier (MPC). In the TCA cycle, acetyl-CoA and oxaloacetate are catalyzed by citrate synthase (CS) to form citrate, which is then converted to isocitrate by aconitase (ACO). Isocitrate undergoes successive oxidative decarboxylation reactions catalyzed by isocitrate dehydrogenase (IDH) and the α-ketoglutarate dehydrogenase complex (α-KGDHC) to generate α-ketoglutarate. Subsequent reactions involve succinyl-CoA synthetase (SCS, which produces ATP via substrate-level phosphorylation), succinate dehydrogenase (SDH, associated with the inner mitochondrial membrane), fumarase (FH), and malate dehydrogenase (MDH). These enzymes sequentially mediate dehydrogenation, hydration, and oxaloacetate regeneration, enabling the cyclic turnover of the TCA cycle (39). In fatty acid metabolism, glucose-derived intermediates or exogenous fatty acids (taken up via CD36) contribute to fatty acid synthesis. Acetyl-CoA serves as a key precursor: it is carboxylated to malonyl-CoA by acetyl-CoA carboxylase (ACC), and then undergoes a series of condensation, reduction, dehydration, and reduction reactions catalyzed by fatty acid synthase (FAS) to form palmitic acid (a long-chain fatty acid) (40). For cholesterol synthesis, acetyl-CoA also acts as the initial substrate. Through the mevalonate pathway, acetyl-CoA is converted to mevalonate by 3-hydroxy-3-methylglutaryl-CoA synthase (HMGS), and then to mevalonate-5-phosphate by mevalonate kinase (MVK). Mevalonate is further processed—with 3-hydroxy-3-methylglutaryl-CoA reductase (HMGCR) acting as a rate-limiting enzyme—to form squalene and ultimately cholesterol (41). Additionally, glutamine, transported into cells via SLC1A5, participates in metabolic processes by replenishing TCA cycle intermediates. Transporters such as GLUT (for glucose uptake) (42), MCT1/4 (for lactate efflux) (43), and LDLR (for cholesterol uptake) (41) regulate the influx and efflux of corresponding metabolites, thereby orchestrating the integrated metabolic network within the cell. Red arrows indicate genes with altered expression in colorectal cancer, highlighting metabolic pathway perturbations relevant to disease progression.

The first and rate-limiting step of fatty acid synthesis is catalyzed by acetyl-CoA carboxylase (ACC), which irreversibly converts cytosolic acetyl-CoA to malonyl-CoA (82) (**Figure 1**). Mitochondrial acetyl-CoA primarily derives from pyruvate dehydrogenase-mediated oxidative decarboxylation, with additional sources including fatty acid β-oxidation, catabolism of branched-chain amino acids (leucine/isoleucine), ketone body breakdown (e.g., β-hydroxybutyrate), and ATP-dependent acetate activation (83). Cytosolic acetyl-CoA is predominantly generated via citrate shuttle-mediated mitochondrial export, where ATP-citrate lyase (ACLY) cleaves citrate into oxaloacetate and acetyl-CoA (84, 85). Under hypoxia or mitochondrial dysfunction, citrate can alternatively be produced through reductive carboxylation of α-ketoglutarate by mitochondrial (IDH2) or cytosolic (IDH1) isocitrate dehydrogenases (86, 87), or imported via the SLC13A5 citrate transporter (88). Another major source of cytosolic acetyl-CoA involves acyl-CoA synthetase short-chain family member 2 (ACSS2)-mediated acetate conversion, with acetate originating from the gut microbiota, histone deacetylation, hydrolyzed acetylated metabolites, or direct pyruvate conversion (89, 90).

FASN catalyzes the elongation cycle by sequentially adding malonyl-CoA units to acetyl-CoA (with NADPH as the reductant) to generate palmitate (a 16-carbon saturated fatty acid) (82). Both ACC and FASN are upregulated in various cancers, including CRC, lung cancer and breast cancer (82, 91). Research has shown that the stage of colorectal cancer is positively correlated with high expression of FASN (92), and the high expression of FASN promotes glycolysis and fatty acid oxidation in colorectal cancer cells (93). Despite the apparent paradox of concurrent fatty acid synthesis and oxidation, FASN-driven lipogenesis provides essential energy substrates—particularly during metastasis, where FAO-derived ATP fuels dissemination (94). Intestinal tissue stem cells, specifically LGR5+ stem cells, are crucial for intestinal maintenance, regeneration, and repair. Loss of adenomatous polyposis (APC) leads to excessive activation of LGR5+ stem cells, resulting in adenocarcinoma formation, indicating that LGR5+ intestinal stem cells (ISCs) are an important source of tumor cells (95). In colitis-associated CRC, ACC1 inhibition suppresses tumorigenesis by impairing Lgr5^+^ intestinal stem cell function (96).

Following palmitate synthesis, elongation by ELOVL enzymes produces stearate and longer-chain saturated fatty acids (SFAs) (40), whereas stearoyl-CoA desaturase-1 (SCD1) generates monounsaturated fatty acids (MUFAs; palmitoleate/oleate) to maintain membrane fluidity. The balance among SFAs, MUFAs, and polyunsaturated fatty acids (PUFAs) is critical for lipid homeostasis, among them essential PUFAs (α-linolenic/linoleic acids) require dietary intake and extracellular uptake before elongation/desaturation by FADS/ELOVLs into arachidonic acid and other PUFAs (40). In CRC, SCD1 overexpression promotes proliferation (97), metastasis (98, 99), and chemoresistance (100), whereas intestinal SCD1 knockout in mice exacerbates inflammation and tumorigenesis—phenotypes reversible by oleate supplementation (101). By converting SFAs to MUFAs, SCD1 alleviates lipotoxicity and reduces PUFA-mediated lipid peroxidation (80), and its inhibition sensitizes CRC cells to RSL3-induced ferroptosis (102). During chemoradiotherapy for colorectal cancer patients, nutritional supplementation with N-3 PUFAs can improve nutritional status and inflammatory indicators, as well as reduce the risk of chemotherapy-induced peripheral neuropathy (103).

SREBP1 (sterol regulatory element-binding protein 1) acts as a master transcriptional regulator of lipogenesis and is overexpressed in multiple cancer types, including prostate cancer (104), colorectal cancer (105), and glioblastoma (106). Its activation upregulates the expression of key lipogenic genes (e.g., FASN, ACLY, ACC, and SCD1) and promotes cell proliferation, migration, invasion, metastasis, and chemoresistance (107). Dysregulation of SREBP1, which leads to excessive lipid production, is a hallmark of tumors. The activity of SREBP1 is hierarchically regulated by the PI3K–AKT–mTOR signaling pathway. Specifically, PI3K-AKT-mTORC1 regulates SREBP1 transcription, mRNA stability, translation, processing and nuclear translocation, nuclear activity, and stability through a series of cascade reactions (107). In colorectal cancer, although SREBP1 mRNA levels are not significantly elevated, activation of the AKT-mTOR pathway may increase the nuclear accumulation of SREBP1 proteins. Suppressing SREBP1 activity via AKT-mTOR inhibition not only inhibits lipogenesis but also promotes ferroptosis in CRC cells (108). Similarly, the knockdown of SREBP1 or its downstream target SCD1 enhances the sensitivity of CRC cells to ferroptosis inducers (102). Furthermore, in colorectal cancer, activator protein-1 (AP-1) (109), AMPK (110), high mobility group A1 (HMGA1) (111), YAP/TAZ signaling (112), TGFβ1 (113), and HIF1α (114) can regulate lipid synthesis through SREBP1.

Obesity is an important risk factor for colorectal cancer (115). Studies have shown that colorectal cancer cells exhibit “fatty acid addiction”: they preferentially metastasize to adipocyte-rich tissues and absorb exogenous fatty acids to fuel FAO (116). As a major energy source alongside glucose/glutamine, FAO generates twice the ATP of carbohydrate oxidation through cyclic β-oxidation, which shortens fatty acids by two carbons per round, yielding acetyl-CoA (for the TCA cycle) and NADH/FADH_2_ (for the ETC) (117). While short-chain fatty acids diffuse freely into mitochondria, long-chain fatty acids require the carnitine shuttle system, regulated by rate-limiting carnitine palmitoyltransferase 1 (CPT1) (118, 119). Notably, fatty acid synthesis and degradation are generally mutually exclusive, as malonyl-CoA generated in the first step of fatty acid synthesis inhibits the activity of CPT1 (117), which controls the first and rate-limiting step of FAO and is regarded as a key regulator in cancer cells. In CRC, valosin-containing protein (VCP) upregulates CPT1 expression by accelerating HDAC1 degradation to enhance FAO and tumor progression (120), while CPT1A-mediated FAO suppresses anoikis to facilitate metastasis (121).

The expression of genes involved in lipid absorption and catabolism is primarily regulated by the PPAR (peroxisome proliferator-activated receptor) family. This family includes three subtypes with distinct tissue expression profiles: PPARα is highly expressed in the liver, kidneys, intestine, heart, skeletal muscle, and brown adipose tissue; PPARβ/δ is widely distributed across tissues, with the highest levels in colonic epithelial cells, skin, and adipocytes; and PPARγ is predominantly expressed in the kidneys, intestinal mucosa, and white/brown adipose tissue (122). Functionally, PPARα enhances fatty acid uptake, esterification, and mitochondrial β-oxidation by upregulating fatty acid transporters (e.g., CD36 and FATP1) and promoting CPT1-mediated mitochondrial fatty acid import. PPARγ collaborates with PPARα in regulating fatty acid oxidation while also participating in adipogenesis, lipid droplet storage, and insulin sensitivity enhancement. PPARδ plays dual roles in promoting both lipid and glucose metabolism (123). PPARα exerts tumor-suppressive effects: its intestinal deficiency promotes colon carcinogenesis in mice by increasing DNMT1-mediated P21 methylation and PRMT6-mediated p27 methylation (124). PPARγ plays dual roles in tumorigenesis: in the AOM/DSS-induced intestinal cancer model, astragaloside IV activates epithelial PPARγ to attenuate inflammation-induced DNA damage, thereby suppressing colon tumor formation (124); conversely, ZDHHC6 promotes colon tumorigenesis by palmitoylating and stabilizing PPARγ to enhance lipid biosynthesis (125). In mouse models, elevated PPARβ/δ accelerates colon tumorigenesis (126), and a high-fat diet further promotes the formation and growth of metastatic liver tumors via PPARβ/δ activation (127). These insights into fatty acid metabolic reprogramming and its regulatory networks in colorectal cancer thus provide a comprehensive understanding.

### 4.4 Cholesterol metabolism: dysregulation, synthesis, and intracellular trafficking

Cholesterol metabolic reprogramming in colorectal cancer orchestrates tumor proliferation, metastasis, and immune evasion through remodeling networks of biosynthesis, exogenous uptake, esterification storage, efflux, and oxidative modification. Epidemiological studies have linked dietary cholesterol intake to elevated gastrointestinal cancer risk (128). Normal tissues maintain cholesterol homeostasis via balanced absorption, synthesis, storage, and export: dietary cholesterol, absorbed as chylomicrons in the small intestine, reaches the liver, which integrates endogenous and exogenous cholesterol for VLDL secretion into circulation; LDL delivers cholesterol to peripheral cells, while HDL mediates reverse transport to the liver/gut for recycling/elimination or to steroidogenic organs (129).

Cholesterol synthesis begins with acetyl-CoA in the cytosol. Two molecules of acetyl-CoA condense to form HMG-CoA, which is then reduced to mevalonate (MVA) by the rate-limiting enzyme HMGCR with NADPH consumption, and subsequently converted to cholesterol in the endoplasmic reticulum via squalene and lanosterol—an energy-intensive process requiring ATP and NADPH (41) (**Figure 1**). Sterol regulatory element-binding protein 2 (SREBP2) acts as the master transcriptional regulator (129): cholesterol depletion triggers the translocation of SCAP-SREBP2 complex to the Golgi, where proteolytic cleavage releases nuclear SREBP2 (nSREBP2) to upregulate cholesterol synthesis genes (e.g., HMGCR, SQLE), cholesterol sufficiency retains the complex in the ER via SCAP-INSIG binding (130). Unlike normal enterocytes, which primarily rely on dietary feedback regulation, CRC cells activate SREBP2-driven biosynthesis to fuel malignant progression, thereby enhancing cell proliferation, invasion, and metabolic reprogramming (41, 131). This metabolic reprogramming is particularly relevant to liver metastasis—the most common site of colorectal cancer metastasis—owing to the liver’s rich portal venous and arterial blood supply, with up to 30–50% of patients developing liver metastases during the disease course (132). During the process of metastasis, circulating tumor cells must integrate intrinsic cellular modifications with external microenvironmental signals to adapt to dynamic conditions in response to environmental stress (133). Notably, hyperactivation of the cholesterol synthesis pathway facilitates CRC liver metastasis, where hepatocyte growth factor (HGF) in the hepatic niche activates PI3K/AKT/mTOR-SREBP2 signaling (134). Intestinal stem cells (ISCs), which maintain epithelial regeneration through continuous self-renewal and differentiation (approximately 5 days per cycle), at crypt bases exhibit cholesterol-dependent hyperproliferation and enhanced self-renewal upon excessive dietary cholesterol or increased endogenous cholesterol synthesis driven by SREBP2, disrupting crypt homeostasis to promote tumorigenesis (135, 136).

Beyond *de novo* synthesis in the endoplasmic reticulum, cancer cells augment exogenous uptake via the LDL receptor (LDLR) and scavenger receptor SR-B1 to acquire LDL and HDL cholesterol, respectively (41). In colorectal cancer, LDLR (137) and SR-B1 (138) are synchronously overexpressed, which enhances the uptake of exogenous cholesterol in the microenvironment to meet the demand for membrane structure during rapid tumor proliferation (139). Cholesterol efflux is the most important step in reverse cholesterol transport. Cholesterol efflux is mediated by ABC transporters (ABCA1, ABCG1, ABCG5, ABCG8) (129). Among them ABCA1 is an integral transporter with widespread expression throughout the body, promoting net cholesterol efflux from peripheral cells to lipid-poor apoA-I for HDL formation (140). ABCG1, a half-size transporter, can dimerize with another ABCG1 or ABCG4 to form a functional transporter; it is highly expressed in diverse cell types including macrophages, but exhibits low expression in hepatocytes and is absent in enterocytes (141). ABCG1 mediates cholesterol efflux to various extracellular acceptors, such as HDL, LDL, albumin, methyl-β-cyclodextrin, and liposomes (129). Studies have demonstrated high ABCG1 expression in human colorectal cancer is correlated with poor patient prognosis (142). Beyond cholesterol, ABCG1 also transports oxysterols (e.g., C7-oxidized oxysterols and 25-hydroxycholesterol) and choline phospholipids (e.g., sphingomyelin) (143, 144). ABCG5 and ABCG8, localized in hepatocytes and enterocytes, are responsible for excreting excess cholesterol from the body into the intestinal lumen or via bile into the intestine (145).

The influx and efflux of cholesterol are regulated by liver X receptors (LXRs), which consist of LXRα and LXRβ isoforms. LXRα is highly expressed in metabolically active tissues and cell types, including the liver, intestine, adipose tissue, and macrophages, whereas LXRβ exhibits broad tissue distribution (146). LXRs form obligate heterodimers with Retinoid X Receptor-α (RXRα; also termed retinoic acid receptor) and activate downstream gene transcription by binding to LXR response elements (LXREs). LXR activation promotes reverse cholesterol transport through multiple mechanisms: it induces the expression of ABCA1 and ABCG1 to facilitate cholesterol efflux from peripheral tissues (e.g., macrophages) to apolipoprotein A-I (apoA-I) for HDL formation; upregulates ABCG5/ABCG8 to enhance biliary cholesterol excretion in hepatocytes and sterol efflux in enterocytes; suppresses NPC1L1 expression to reduce intestinal cholesterol absorption; and inhibits SREBP-1c activity to downregulate genes involved in fatty acid and triglyceride synthesis (147). In colitis models, LXR activation increases colonic crypt cell proliferation and colon length, indicating its pro-regenerative role in response to intestinal injury (148). Conversely, in CRC LXR activation suppresses the proliferation of human colorectal cancer cells and the growth of murine intestinal tumors through regulating cholesterol metabolism (148, 149). Collectively, these findings underscore the critical role of cholesterol metabolic reprogramming in colorectal cancer pathogenesis, highlighting its potential as a promising target for therapeutic intervention.

### 4.5 Glutamine metabolism: addiction and fuel for tumor progression

Glutamine metabolic reprogramming in colorectal cancer establishes a core “glutamine addiction” phenotype, driving malignant progression and therapy resistance through dynamic regulation of the carbon/nitrogen supply, redox homeostasis, and epigenetic control. As the most abundant amino acid in humans, glutamine functions as a nitrogen carrier for the biosynthesis of non-essential amino acids, purines, pyrimidines, and fatty acids, while contributing to the TCA cycle via α-ketoglutarate (α-KG) (150, 151). Its plasma concentration (600–700 μM) exceeds that of other amino acids by nearly an order of magnitude, reflecting high physiological demand (152).

**Figure 2:**
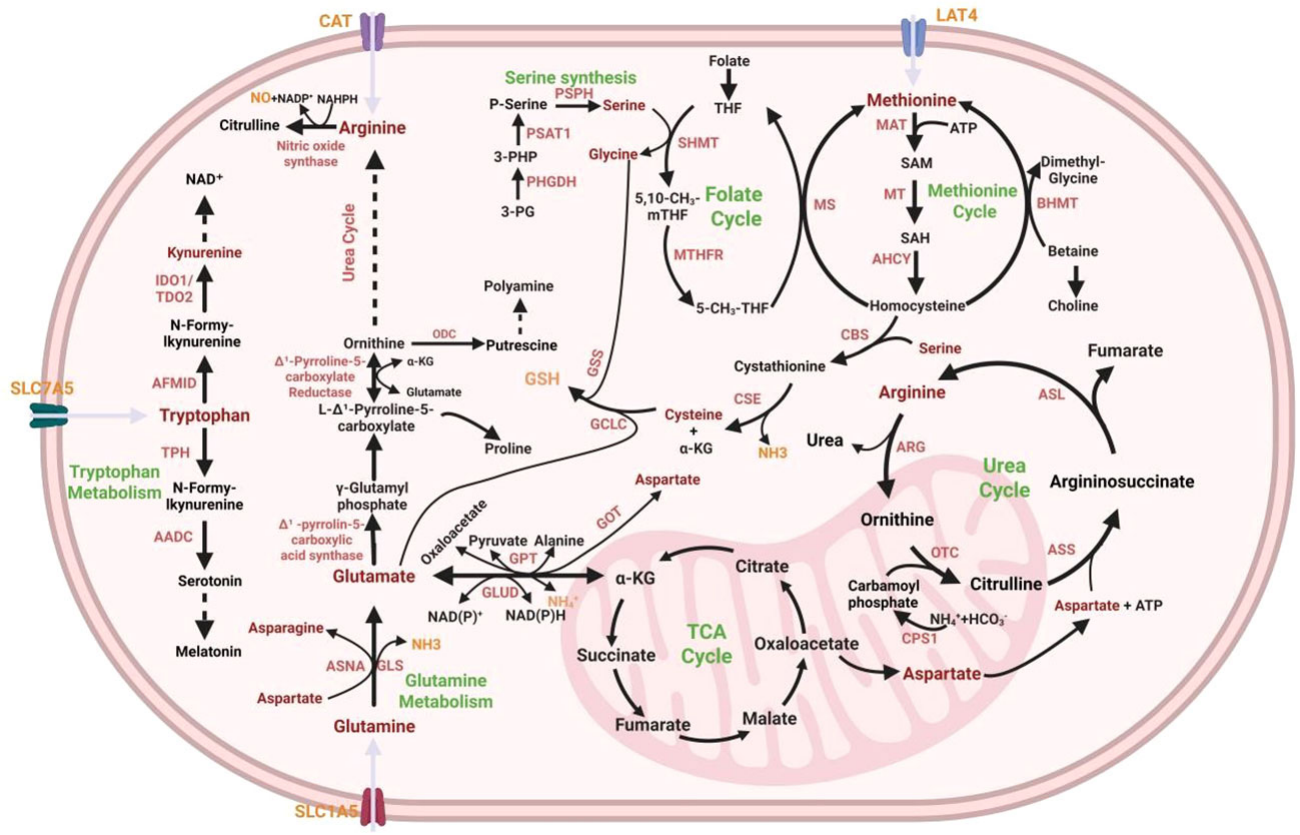
Amino acid metabolism in in colorectal cancer cells. Glutamine, transported by SLC1A5, converts to glutamate, feeding into the TCA cycle as α-ketoglutarate (α-KG), supporting arginine biosynthesis (via citrulline) and proline production (153). Key enzymes like glutaminase (GLS) drive glutamine to glutamate conversion (153). Serine synthesis from 3-phosphoglycerate involves phosphoserine aminotransferase 1 (PSAT1) and phosphoserine phosphatase (PSPH), feeding the folate cycle via serine hydroxymethyltransferase (SHMT) to generate one carbon units for methionine regeneration (via methionine synthase, MS) and S-adenosylmethionine (SAM) mediated methylation (172). Arginine biosynthesis links to the urea cycle, where enzymes like arginase (ARG) and ornithine transcarbamylase (OTC) detoxify ammonia, balancing nitrogen metabolism (173). Tryptophan (Trp), imported by SLC7A5, follows the kynurenine pathway (via IDO1/TDO) for immune regulation or the serotonin pathway (via TPH) for neurotransmitter production (174). Polyamines (from ornithine via ornithine decarboxylase, ODC) regulate proliferation, while proline contributes to collagen and redox balance (173). This network highlights the interdependencies of amino acid, energy, and biosynthetic pathways in cellular function.

Glutaminolysis—the catabolic breakdown of glutamine—is essential for proliferating cells to replenish TCA cycle intermediates. Membrane-bound glutamine transporters (SLC1A5, SLC38A1/2, SLC6A14) mediate cellular uptake, then a mitochondrial variant of SLC1A5 delivers glutamine to the mitochondrial matrix, where glutaminase (GLS) converts it to glutamate. Subsequent transformations by GLUD1, GOT2, or GPT2 yield α-KG while releasing ammonia, aspartate, or alanine, respectively (153) (**Figure 2**). The TCA cycle functions as a central hub for metabolite interconversion across multiple metabolic pathways. In rapidly proliferating cancer cells, the high turnover rate of metabolites necessitates reliance on glutamine anaplerosis (153). In colorectal cancer, the overexpression of glutamine transporters, SLC1A5 (154), SLC38A1/2 (155), and SLC6A14 (156), drives enhanced glutamine uptake—a key metabolic adaptation that enables CRC cells to survive and proliferate in the nutrient-limited tumor microenvironment. Notably, while the levels of various amino acids—including glutamate, aspartate, and alanine—are markedly elevated in CRC tissues, glutamine concentrations remain nearly comparable between CRC and normal intestinal tissues, reflecting robust glutaminolysis in colorectal cancer (157). Glutaminase 1 (GLS1), the rate-limiting enzyme in glutaminolysis, is transcriptionally regulated by c-Myc, HIF1α, and the Wnt/β-catenin signaling pathway, which are critical drivers of colorectal cancer (158, 159). Knockout of GLS1 in CRC activates ROS-related signaling pathways, increasing tumor immunogenicity via enhanced immunoproteasome activity and anti-tumor immunity activation, leading to reduced tumor burden in CRC models (160). During anaplerosis, mitochondrial GLUD1 catalyzes critical glutamate-to-α-KG conversion, with released ammonia modulating autophagy and intracellular pH (161, 162). CRC resistance to glucose deprivation correlates with GLUD1 and SLC25A13 (mitochondrial aspartate-glutamate carrier) levels, and their co-expression predicts aggressive phenotypes and poor prognosis (163). Cytosolic GPT1 and mitochondrial GPT2 generate both α-KG and alanine for protein synthesis (164), among them GPT2 is particularly critical in KRAS-driven CRC, breast cancer, and glioblastoma, as it sustains mitochondrial metabolic homeostasis and fuels anabolic metabolism to support the aggressive proliferation of KRAS-mutant tumor cells (153).

As cancer cells poorly absorb exogenous aspartate, GOT1/GOT2 support tumorigenesis by providing cytosolic aspartate (a precursor for nucleotide/protein synthesis) while constituting the malate-aspartate shuttle—facilitating NADH transfer across membranes to maintain redox balance (165). KRAS-mutant CRC cells exhibit reduced aspartate but elevated asparagine levels, adapting to glutamine deprivation via PI3K-AKT-mTOR-mediated activation of asparagine synthetase (ASNS) (166). Notably, KRAS-driven glutamine metabolic rewiring is context-dependent: in pancreatic ductal adenocarcinoma (PDAC) models, mutant KRAS suppresses GLUD1 while upregulating GOT1 (167), whereas in CRC its expression remains unaltered.

As the Warburg effect blocks pyruvate from being converted to acetyl-CoA, glutamine-derived α-ketoglutarate (α-KG) replenishes TCA cycle intermediates and acts as a primary carbon source to sustain citrate synthesis for *de novo* fatty acid synthesis (168). Notably, glutaminolysis confers dual bioenergetic value: in addition to contributing carbon skeletons, the ammonia nitrogen generated during its deamination directly supports *de novo* purine and pyrimidine biosynthesis. Under hypoxia, cells upregulate glutamine uptake—rather than accumulating glutamine metabolites, nucleotide precursors such as dihydroorotic acid and inosine monophosphate (IMP) accumulate, while glutamine-derived aspartate is incorporated into pyrimidine nucleotides (169). *De novo* pyrimidine synthesis initiates with carbamoyl phosphate formation, and *de novo* purine synthesis begins with the binding of phosphoribosyl pyrophosphate (PRPP) to glutamine. Glutamine deficiency induces S-phase cell cycle arrest due to nucleotide depletion, and this arrest can be reversed by supplementing with aspartate (170). Additionally, glutamine is critical for redox regulation: its metabolism maintains the balance of ROS primarily via two pathways. The first involves synthesizing glutathione, a key intracellular antioxidant; the second entails GLUD-mediated NADPH production, which supplies reducing equivalents to glutathione (168). In PIK3CA-mutant colorectal cancer, GPT2 upregulation drives glutamine dependency. Glutaminase inhibition then suppresses cancer cell growth, elevates intracellular ROS, and concurrently upregulates uridine phosphorylase 1 (UPP1), which promotes 5-FU conversion to its active form and thereby enhances 5-FU efficacy (171).

Thus, glutamine metabolic reprogramming in colorectal cancer—encompassing its role as a carbon/nitrogen source, redox regulator, and mediator of therapeutic responses—represents a pivotal metabolic hallmark that links “glutamine addiction” to malignant progression and therapy resistance, with implications for understanding CRC pathogenesis.

### 4.6 Methionine metabolism: methyl donor supply and epigenetic regulation

Methionine (Met) metabolic reprogramming in colorectal cancer underpins epigenetic remodeling, redox homeostasis, and the acquisition of metastatic and drug-resistant phenotypes. Dysregulation of the methionine cycle and folate-mediated one-carbon metabolism drives widespread aberrations in DNA/RNA/protein methylation, endowing cancer cells with proliferative advantages and immune evasion capabilities. As an essential sulfur-containing amino acid, methionine cannot be synthesized *de novo* in humans and must be acquired through dietary intake; its metabolism involves a series of catabolic and recycling reactions collectively termed the methionine cycle.

The methionine cycle initiates with the conversion of methionine to S-adenosylmethionine (SAM) via methionine adenosyltransferase (MAT), the rate-limiting enzyme. SAM, the universal methyl donor, is demethylated by methyltransferases (MTs) to form S-adenosylhomocysteine (SAH), which is then hydrolyzed by S-adenosylhomocysteine hydrolase (AHCY) into homocysteine (Hcy). Hcy is subsequently remethylated to regenerate methionine via either methionine synthase (MS, requiring vitamin B12 as a cofactor) or betaine-homocysteine methyltransferase (BHMT), completing the cycle (**Figure 2**).

MAT exists in three tissue-specific isoforms:MAT1A (encoding the α1 subunit) forms dimers (MAT III) or tetramers (MAT I), predominantly expressed in the liver as a marker of differentiated hepatocytes; MAT2A (encoding the α2 subunit) forms tetramers (MAT II), widely expressed in extrahepatic tissues and fetal liver (gradually replaced by MAT1A during development) and associated with rapid proliferation and dedifferentiation (175, 176). In CRC, the tumor-to-normal tissue ratio of MAT activity correlates with disease progression (177). Elevated MAT2A and SAM levels are observed in murine colon cancer models and polyps, where growth factors (EGF, IGF-1, leptin) upregulate MAT2A to promote CRC cell growth (178). Paradoxically, endogenous SAM accumulation or exogenous SAM treatment inhibits CRC proliferation, induces apoptosis (178, 179), and reverses promoter hypomethylation of oncogenes such as c-Myc and Ras (180). SAM differentially regulates MAT isoforms: it sustains MAT1A expression but suppresses MAT2A, creating a negative feedback loop that limits excessive MAT2A-driven SAM production (181).

As the universal methyl donor in cells, SAM transfers methyl groups to the proteome or DNA via methyltransferases before being converted to SAH. Methionine metabolism influences epigenetic regulation through SAH, thereby impacting tumor initiation and progression. The characteristic hypermethylation within promoter regions of colorectal cancer tumor suppressor genes indicates that the epigenome likely plays a key role in mediating non-genetic sequence modifications driving malignant phenotype (182). Chromosomal DNA methylation (catalyzed by DNA methyltransferases [DNMTs]), histone methylation (catalyzed by histone methyltransferase families), and RNA methylation (catalyzed by N-adenosyl methyltransferases) are all regulated by intracellular SAM levels (183). In CRC, methionine supplementation can suppress PCSK9 expression and chemoresistance via DNMT1-mediated hypermethylation of the Sirtuin 6 promoter (184). In parallel, reduced SAM levels decrease METTL16-mediated methylation of MAT2A mRNA, enhancing MAT2A translation to restore SAM homeostasis (185). Global DNA hypomethylation—an early event in CRC tumorigenesis linked to proto-oncogene activation—may reflect SAM dysregulation (186), though this relationship remains incompletely characterized.

Other SAM-consuming methyltransferases, including phosphatidylethanolamine N-methyltransferase (PEMT) and nicotinamide N-methyltransferase (NNMT), further modulate SAM pools (187). NNMT acts as a negative regulator of intracellular SAM: its overexpression in CRC cells reduces SAM and global methylation while elevating SAH, thereby increasing the sensitivity of CRC cells to OXPHOS inhibitors (188). In cancer-associated fibroblasts, NNMT-mediated depletion of SAM pools diverts methyl groups from DNA/histone methylation, promoting metastasis (189). These methyltransferases, with distinct Km values and SAM affinities, collectively maintain SAM homeostasis across concentration gradients.

SAH cleavage yields Hcy and adenosine, Hcy remethylation to methionine requires 5-methyltetrahydrofolate (5-MTHF) as a methyl donor. 5-MTHF regeneration depends on the folate cycle: dihydrofolate reductase (DHFR) reduces dihydrofolate (DHF) to tetrahydrofolate (THF); serine hydroxymethyltransferase (SHMT) transfers a one-carbon unit to THF, forming 5,10-methylene-THF; and methylenetetrahydrofolate reductase (MTHFR) irreversibly reduces this to 5-MTHF. Methionine synthase (MS) then transfers the methyl group from 5-MTHF to Hcy, regenerating methionine and THF (172). Studies have demonstrated that low folate intake disrupts the folate-methionine cycle, inducing global DNA hypomethylation of oncogenes and tumor suppressor genes, thereby elevating the risk of colorectal cancer (190).

Serine metabolism intersects with methionine/folate cycles as the primary source of one-carbon units for THF regeneration. Serine biosynthesis originates from the glycolytic intermediate 3-phosphoglycerate (3-PG): phosphoglycerate dehydrogenase (PHGDH) oxidizes 3-PG to 3-phosphohydroxypyruvate (3-PHP), generating NADH; phosphoserine aminotransferase (PSAT) transaminates 3-PHP to 3-phosphoserine (3-PS); and phosphoserine phosphatase (PSPH) hydrolyzes 3-PS to serine. Phosphoglycerate dehydrogenase (PHGDH) is a key enzyme in *de novo* serine biosynthesis. It oxidizes 3-phosphoglycerate (3-PG) to generate serine and glycine, thereby diverting glycolytic flux to rapidly produce metabolites necessary for robust anabolism. In CRC, PHGDH is markedly upregulated: Cullin 4A (Cul4A)-mediated PHGDH ubiquitination enhances its stability and activity, elevating SAM levels to promote metastasis via SETD1A-dependent histone methylation of cell adhesion genes (191). As a direct consequence of this enhanced PHGDH activity, murine models demonstrate that augmented serine biosynthesis fuels glutamine utilization to drives intestinal tumorigenesis (192). Mitochondrial serine metabolism supports purine nucleotide synthesis, conferring 5-FU resistance by mitigating DNA damage, conversely, serine deprivation sensitizes CRC to 5-FU (193). Additionally, studies have demonstrated that age-related mitochondrial DNA mutations can accelerate intestinal tumorigenesis through upregulation of the serine biosynthesis pathway (194).

Beyond methylation, SAM-derived metabolites enter the transsulfuration pathway, where cystathionine β-synthase (CBS) transfers sulfur from Hcy to serine, forming cystathionine and ultimately cysteine. Transsulfuration flux is governed by the SAM/SAH ratio: high SAM activates CBS while inhibiting MTHFR, whereas low SAM inactivates CBS to favor remethylation (172, 183). SAM levels also modulate mTOR signaling: elevated SAM promotes PP2A methylation to activate mTORC1, while low SAM inhibits mTORC1 and induces autophagy for nutrient recycling (187). Taken together, these insights highlight the multifaceted role of methionine metabolic reprogramming in colorectal cancer biology, emphasizing its potential as a target for therapeutic intervention and biomarker development.

### 4.7 Tryptophan metabolism: kynurenine pathway and metabolic flux regulation

Tryptophan (Trp) metabolic reprogramming in colorectal cancer drives tumor immune evasion, metastatic colonization, and therapy resistance through microenvironmental Trp depletion, generation of immunosuppressive metabolites, and crosstalk with microbiota-derived metabolites. Synergistic overactivation of the oncogenic kynurenine (Kyn) pathway and gut microbial Trp catabolism represents a pivotal mechanism underlying malignant progression. As an essential amino acid, L-tryptophan is exclusively obtained from dietary intake, with its free pool regulated by nutritional uptake and flux through three major metabolic pathways.

Over 95% of free Trp is degraded via the kynurenine pathway, initiated by oxidative cleavage of Trp to N-formylkynurenine—catalyzed by tryptophan-2,3-dioxygenase (TDO) or indoleamine-2,3-dioxygenase (IDO) (174). N-formylkynurenine is then hydrolyzed to kynurenine by formamidase (AFMID). Subsequent metabolism bifurcates into two branches: (i) Kynurenine is converted to 3-hydroxykynurenine by kynurenine-3-monooxygenase (KMO), then to 3-hydroxyanthranilic acid and alanine via kynureninase (KYNU); (ii) Kynurenine is directly converted to kynurenic acid by kynurenine aminotransferase (KAT). The terminal metabolite quinolinic acid is converted to nicotinic acid mononucleotide (NAMN) by quinolinate phosphoribosyltransferase (QPRT) for NAD^+^ synthesis. Pathway flux is controlled by rate-limiting enzymes: IDO1, IDO2 (widely expressed in immune/non-immune cells and regulated by cytokines/inflammation), and TDO (primarily localized in the liver, regulating hepatic conversion of tryptophan) (195) (**Figure 2**).

In CRC, tryptophan (Trp) transporters (SLC7A5, SLC1A5) and key components of the kynurenine (Kyn) metabolic pathway (TDO2, IDO1, AFMID) are significantly upregulated, a molecular adaptation that enhances Trp uptake and catabolism to fuel tumor cell proliferation (196). Among them IDO expression serves as a prognostic biomarker in locally advanced rectal cancer (LARC) patients receiving neoadjuvant chemoradiotherapy (CRT). Meanwhile, its elevated activity further drives tryptophan degradation via the kynurenine pathway, inducing local tryptophan starvation and kynurenine accumulation to impair effector T-cell function and enhance peripheral immune tolerance (197). Consistent with this mechanistic insight, in a murine intestinal cancer model, IDO knockout suppresses tumor initiation and progression. Beyond impairing T-cell immunity, kynurenine and quinolinic acid activate β-catenin, stimulate the proliferation of human colon cancer cells, and accelerate tumor growth in mice (198). Additionally, Kyn pathway metabolites enhance CRC malignancy via PI3K/AKT activation (199). Notably, in APC-mutant CRC, APC deficiency upregulates TCF4/β-catenin-dependent TDO2 transcription, activating the Kyn-AhR axis to boost glycolysis for anabolic growth and CXCL5-mediated macrophage recruitment (200).

Serotonin (5-hydroxytryptamine, 5-HT) represents another major metabolic pathway of tryptophan, with its signaling cascade encompassing the serotonin transporter (SLC6A4), serotonin receptors (HTR), tryptophan hydroxylase (TPH), and monoamine oxidase (201). As a neurotransmitter that regulates mood, sleep, appetite, and physiological functions, 5-HT biosynthesis begins with the rate-limiting hydroxylation of tryptophan catalyzed by TPH—this reaction consumes tetrahydrobiopterin (BH_4_) and generates 7,8-dihydrobiopterin (BH_2_) to form 5-hydroxytryptophan (5-HTP). Subsequent decarboxylation by aromatic L-amino acid decarboxylase (AADC), which requires pyridoxal 5’-phosphate (PLP) as a cofactor, yields 5-HT. Peripheral 5-HT—primarily synthesized by enterochromaffin cell TPH1—regulates gastrointestinal motility and secretion (202). In CRC, 5-HT receptors (HTR1B/D/F) are overexpressed in tumors and Lgr5^+^ cancer stem cells (CSCs), where 5-HT activates Wnt/β-catenin signaling to promote tumorigenesis and CSC self-renewal (203). Meanwhile, Tph2 is significantly upregulated in the core region of colorectal cancer tumors, and conditional knockout of Tph2 in intestinal epithelial cells markedly suppresses tumorigenesis in a murine intestinal cancer model (203). In DSS-induced colitis models, 5-HT also exerts proinflammatory effects: it exacerbates dextran sulfate sodium (DSS)-induced colitis, whereas inhibiting 5-HT biosynthesis or tryptophan-based therapy mitigates these proinflammatory effects (204). In colorectal cancer, patients with multiple metastatic colorectal cancer exhibit serum 5-hydroxytryptamine (5-HT) levels more than twice those of the normal population, with a significant reduction observed following surgical tumor resection. Further experimental evidence confirms that the serotonin transporter (SERT) mediates serotonin uptake into colorectal cancer cells, enhances the expression of YAP-related proteins via the RhoA-ROCK1/2 signaling pathway, and promotes colorectal cancer cell growth both *in vivo* and *in vitro (*205).

The indole pathway, a branch of the tryptophan decarboxylation (TDC) pathway, constitutes the third major metabolic route for tryptophan and is mediated by intestinal microbiota. Within this pathway, TDC—under the influence of gut microbes—catalyzes the decarboxylation of tryptophan to form tryptamine. Tryptamine is then further metabolized into diverse indoleacetic acid derivatives, such as indole acrylic acid, indole-3-acetic acid (IAA), and indole-3-acetaldehyde (206). Dysbiosis suppresses indole production while accumulating Kyn, inactivating aryl hydrocarbon receptor (AhR) signaling to impair epithelial integrity, promote inflammation, and suppress antitumor immunity. Notably, Acinetobacter radioresistens-derived IAA activates AhR/Wnt/β-catenin signaling to maintain intestinal stemness, whereas indole-3-lactic acid (ILA) inhibits STAT3 phosphorylation to suppress HK2-mediated glycolysis in cancer cells (207).

### 4.8 Arginine metabolism: ornithine cycle and nitric oxide (NO)-mediated effects

Arginine (Arg), a semi-essential amino acid, acts as a critical precursor for the synthesis of proteins, polyamines, creatine, and nitric oxide (NO). Its metabolic network plays pivotal roles in colorectal cancer progression by regulating tumor cell proliferation, survival, and immune microenvironment modulation through multiple mechanisms. Dietary Arg restriction or therapeutic depletion via arginine deiminase (ADI)/arginase exerts inhibitory effects on colon cancer (208). Physiologically, Arg is acquired through two pathways: cellular uptake mediated by cationic amino acid transporters (CAT1-3) and the urea cycle. CAT1–3 exhibit differential expression across tissues, Among them CAT1 is highly expressed in tumors such as colorectal (209), breast (210), and ovarian cancers (211). Beyond CAT1, SLC6A14—a protein with low expression in normal tissues—serves as a Wnt target and its expression level is strongly correlated with colorectal cancer (212). High SLC6A14 expression in colorectal cancer promotes cancer cell survival and metastasis by enhancing arginine uptake (156, 213).

The urea cycle commences with the synthesis of carbamoyl phosphate from ammonia and CO_2_ by carbamoyl phosphate synthetase I (CPSI), followed by the condensation of carbamoyl phosphate with ornithine to form citrulline, catalyzed by ornithine transcarbamylase (OTC). Argininosuccinate synthase (ASS) then conjugates citrulline with aspartate to generate argininosuccinate, which is cleaved into Arg and fumarate by argininosuccinate lyase (ASL). Finally, Arg is hydrolyzed by arginase to regenerate ornithine and release urea (173) (**Figure 2**). In normal tissues, intestinal cells convert glutamine/proline to citrulline, which is further used for renal Arg synthesis via ASS1/ASL. By contrast, the ASS1 gene promoter is frequently epigenetically silenced in malignant melanoma, hepatocellular carcinoma, mesothelioma, and prostate cancer (214, 215). Due to reduced ASS1 expression, such tumor cells are highly dependent on extracellular arginine for survival. In colorectal cancer, however, ASS1 expression is upregulated, making these cells independent of exogenous arginine and distinguishing CRC from the aforementioned cancer types (214, 216). Studies have shown that elevated ASS1 expression promotes intestinal epithelial cell regeneration (217), yet its role in colorectal cancer initiation and progression remains controversial (216–218). Loss of ASS1 in the intestine may induce intrinsic compensation via amino acid transporters and extrinsic compensation in distant organs such as the liver (216).

Arginine metabolism intersects with the TCA cycle via aspartate: glutamic-oxaloacetic transaminase (GOT) converts oxaloacetate to aspartate, which fuels the urea cycle through ASS1, while fumarate derived from ASL re-enters the TCA cycle (219). Arginine catabolism also relies on arginase (ARG), which hydrolyzes Arg into urea and ornithine. Arginase exists in two isoforms: ARGI is a cytoplasmic enzyme predominantly expressed in the liver, responsible for converting ammonia into non-toxic urea for excretion; ARGII, found in both the cytoplasm and mitochondria, is widely expressed in tissues such as the kidney, small intestine, prostate, and brain, and can regulate local arginine concentrations while participating in the synthesis of polyamines, proline, glutamate, and nitric oxide (NO) (220). Ornithine either re-enters the urea cycle or contributes to polyamine biosynthesis via ornithine decarboxylase (ODC)—the rate-limiting enzyme that converts ornithine to putrescine, which is one of the initial metabolic intermediates in cytoplasmic polyamine metabolism (173). Ornithine decarboxylase (ODC) is highly expressed in tumors including breast (221), gastric (222), and colorectal cancer (223), and its overexpression-driven excessive polyamine accumulation promotes tumor initiation and metastasis. However, recent studies have demonstrated that low spermine intake and high spermidine intake reduce colorectal cancer risk (224), while another study revealed that spermidine alleviates colitis and lowers colorectal cancer incidence (225). These findings highlight the divergent roles of polyamines in colorectal cancer.

Beyond the urea cycle, Arg is metabolized by nitric oxide synthase (NOS) to produce NO and citrulline (226), competing with arginase for the same substrate, and activation of arginase reduces NOS-mediated arginine utilization, thereby decreasing NO production (227). Enhanced endogenous NO generation by intestinal epithelial cells improves epithelial integrity, alleviates colitis, and mitigates inflammation-associated colon cancer (228). Alternatively, arginine decarboxylase (ADC) converts Arg to agmatine—a competitive inhibitor of NOS that suppresses NO synthesis—and agmatinase further metabolizes agmatine to putrescine, which in turn promotes polyamine biosynthesis (229). Collectively, these findings underscore the intricate and context-dependent roles of arginine metabolism and its downstream metabolites in colorectal cancer pathogenesis, reflecting the complexity of their involvement in tumor initiation, progression, and associated pathological processes.

## 5 Impact of colorectal cancer metabolism on the tumor microenvironment and immunity

### 5.1 Glucose deprivation: metabolic competition and immune cell dysfunction

**Figure 3:**
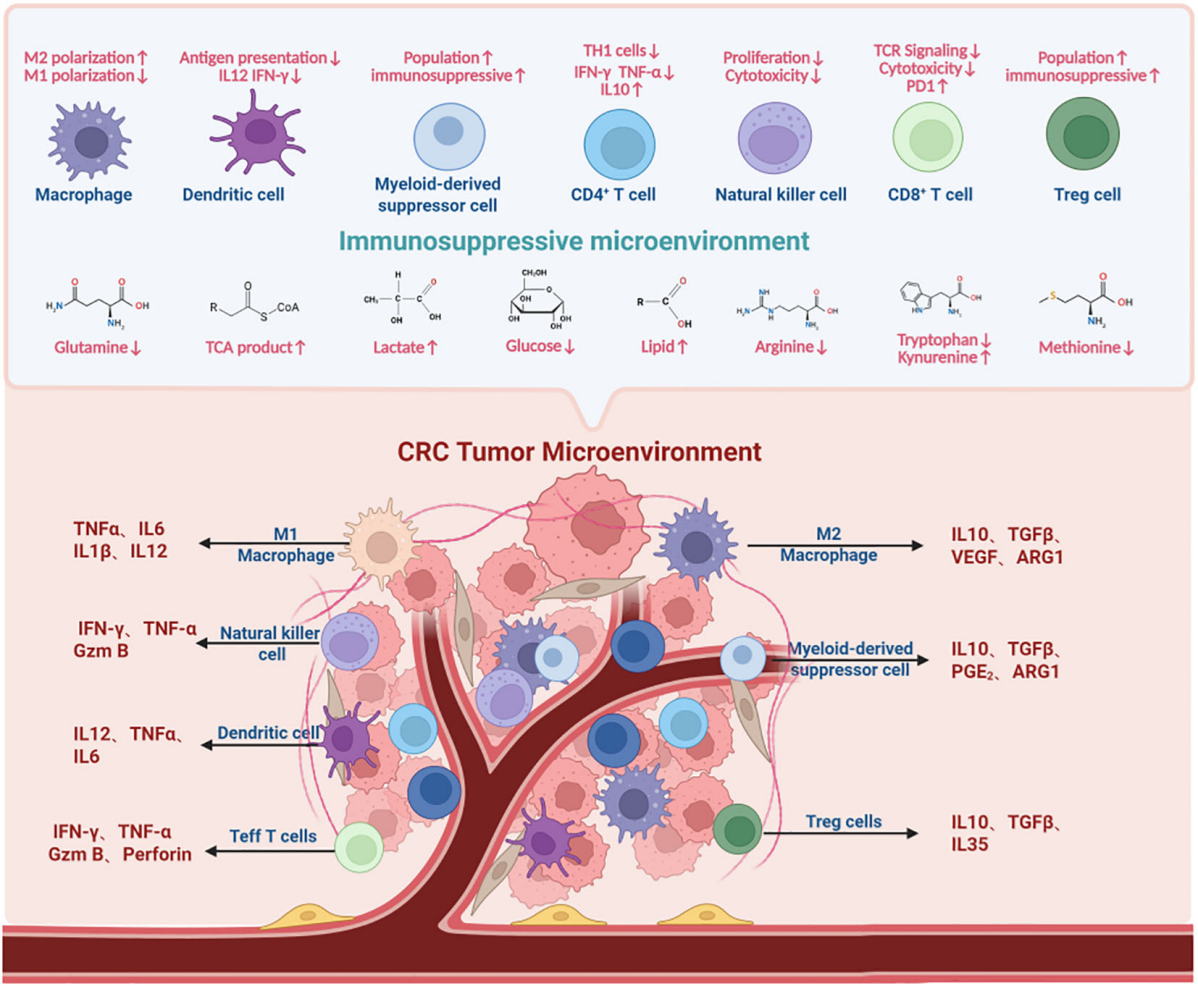
The immunosuppressive network in the colorectal cancer tumor microenvironment. In CRC, decreased levels of glutamine, TCA cycle intermediates, glucose, arginine, tryptophan (shunted to kynurenine), and methionine, coupled with increased lactate, lipids, and kynurenine, impinge on immune cell functions. These metabolic alterations disrupt the normal behavior of various immune cells, including macrophages, dendritic cells, natural killer cells, T cells, and myeloid - derived suppressor cells. Collectively, they shape the immunosuppressive microenvironment, as depicted by the changed phenotypes and functions of immune cells (e.g., M2 polarization of macrophages, impaired antigen presentation by dendritic cells, expanded myeloid-derived suppressor cells, and regulatory T cells, altered activity of T cells and natural killer cells) within the CRC tumor microenvironment.

Within the highly metabolically active tumor microenvironment of colorectal cancer, malignant cells exhibit voracious glucose consumption via the Warburg effect to meet the biosynthetic and bioenergetic demands of rapid proliferation. This leads to profound glucose depletion, imposing severe metabolic stress on infiltrating immune cells. While most effector immune populations rely critically on glucose as their primary energy source and biosynthetic precursor—leaving them functionally impaired under glucose restriction—certain immunosuppressive subsets display metabolic flexibility or even thrive in these conditions, collectively exacerbating immunosuppression within the TME. CRC cells achieve glucose dominance through GLUT1 overexpression and upregulation of glycolytic enzymes, compounded by aberrant vasculature-induced perfusion deficits, establishing steep intratumoral glucose gradients with the lowest concentrations in tumor cores (**Figure 3**). This “glucose-starved” niche directly suppresses key antitumor effectors: cytotoxic T lymphocytes (CTLs/CD8^+^ T cells) and natural killer (NK) cells.

**Figure 4:**
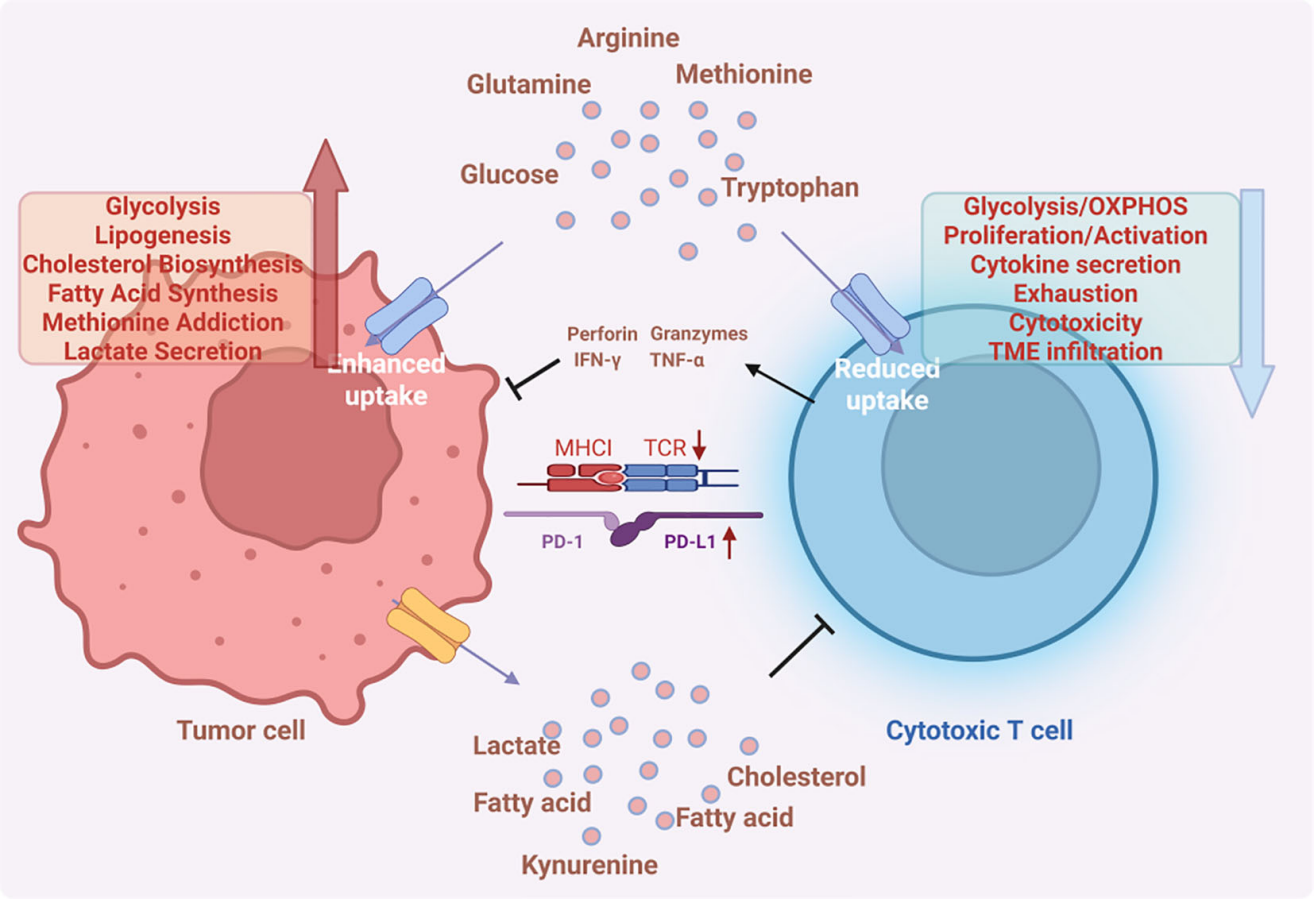
Metabolic and immune interactions in the tumor microenvironment. Tumor cells exhibit enhanced uptake of nutrients like glucose, glutamine, arginine, methionine, and tryptophan, fueling pathways such as glycolysis, lipogenesis, cholesterol biosynthesis, and methionine metabolism, while secreting lactate. They also release metabolites (lactate, fatty acids, cholesterol, kynurenine) that suppress cytotoxic T cells. These T cells show reduced nutrient uptake, and their functions (glycolysis/OXPHOS, proliferation, cytokine secretion, cytotoxicity, TME infiltration) are impaired. Immune checkpoints (PD - 1/PD - L1) further inhibit T cell activity, forming a network where tumor - driven metabolic reprogramming and metabolite - mediated immunosuppression collaborate to evade anti - tumor immune responses.

NK cells—critical innate immune effectors—exert antitumor activity through cytotoxic molecules (IFN-γ, granzymes) and chemokine-mediated T cell recruitment. Activated NK cells upregulate both glycolysis and OXPHOS (230), metabolic suppression of which impair IFN-γ production and cytotoxicity (231). Quiescent T cells rely on oxidative phosphorylation to maintain basal metabolic rates, whereas the CD8^+^ T cell immune response entails a 1000-fold expansion in activated cell numbers relative to the quiescent state (232). This is accomplished via clonal expansion of activated T cells, a process demanding substantial glucose for energy. Like cancer cells, proliferating effector T cells engage in aerobic glycolysis which is critical for sustaining heightened proliferation and producing inflammatory cytokines, and these cytokines are pivotal for preserving anti-tumor immunity and fostering immune memory (233, 234). Even in the presence of glutamine as an alternative fuel source, cytotoxic T cells fail to proliferate in glucose-depleted media (235), while glucose restriction strongly suppresses interferon-γ expression in CD8^+^ T cells (**Figure 4**) (236). Hexokinase 2 (HK2) catalyzes the rate-limiting step in glycolysis, when cellular reactive oxygen species (ROS) reach a threshold level, HK2 undergoes autophagic degradation. In CD8^+^ T cells, NF-κB-inducing kinase (NIK) prevents autophagic degradation of HK2 by regulating cellular ROS levels (237). This preservation of HK2 enhances glycolysis, thereby augmenting anti-tumor immunity in melanoma and colorectal cancer. Like CD8^+^ T cells, CD4^+^ T cell activation depends on glycolysis, glut1 deficiency reduces thymocyte counts, suppresses the expansion of effector CD4^+^ T cells, and impairs cytokine production in various CD4^+^ T cell subsets, including interferon-γ, IL-4, and IL-17 (238).

In stark contrast, immunosuppressive regulatory T cells (Tregs) thrive in glucose-poor TMEs through metabolic adaptations: Foxp3 reprograms Treg metabolism by suppressing Myc/glycolysis while enhancing OXPHOS and NAD^+^ oxidation, rendering them less glucose-dependent and more reliant on FAO (239). This metabolic flexibility allows Tregs to maintain suppressive function in lactate-rich, hypoglycemic niches, while their immunosuppressive capacity is impaired by glucose restoration (240). Myeloid-derived suppressor cells (MDSCs) exhibit metabolic flexibility within the tumor microenvironment. On the one hand, MDSCs possess the capacity to acquire and utilize large quantities of glucose in the TME; their substantially increased glucose consumption generates high-energy nucleotides and carbon intermediates that support their immunosuppressive mechanisms while exacerbating local glucose starvation (241). Glycolytic restriction impairs the suppressive capacity of MDSCs in tumors, 2-deoxyglucose (2-DG), a glycolytic pathway inhibitor, markedly suppresses the differentiation of monocytic MDSCs (M-MDSCs) and their precursors in the TME (242). Meanwhile, methionine enkephalin (MENK), an endogenous opioid peptide, reduces glycolysis and decreases ROS production in MDSCs via the PI3K/AKT/mTOR pathway, thereby inhibiting colon cancer progression (243). On the other hand, lipid accumulation in the TME induces metabolic reprogramming in MDSCs, shifting from glycolysis to FAO, and enables them to utilize oxidized lipids as a primary energy source to enhance their immunosuppressive activity (244, 245).

**Figure 5:**
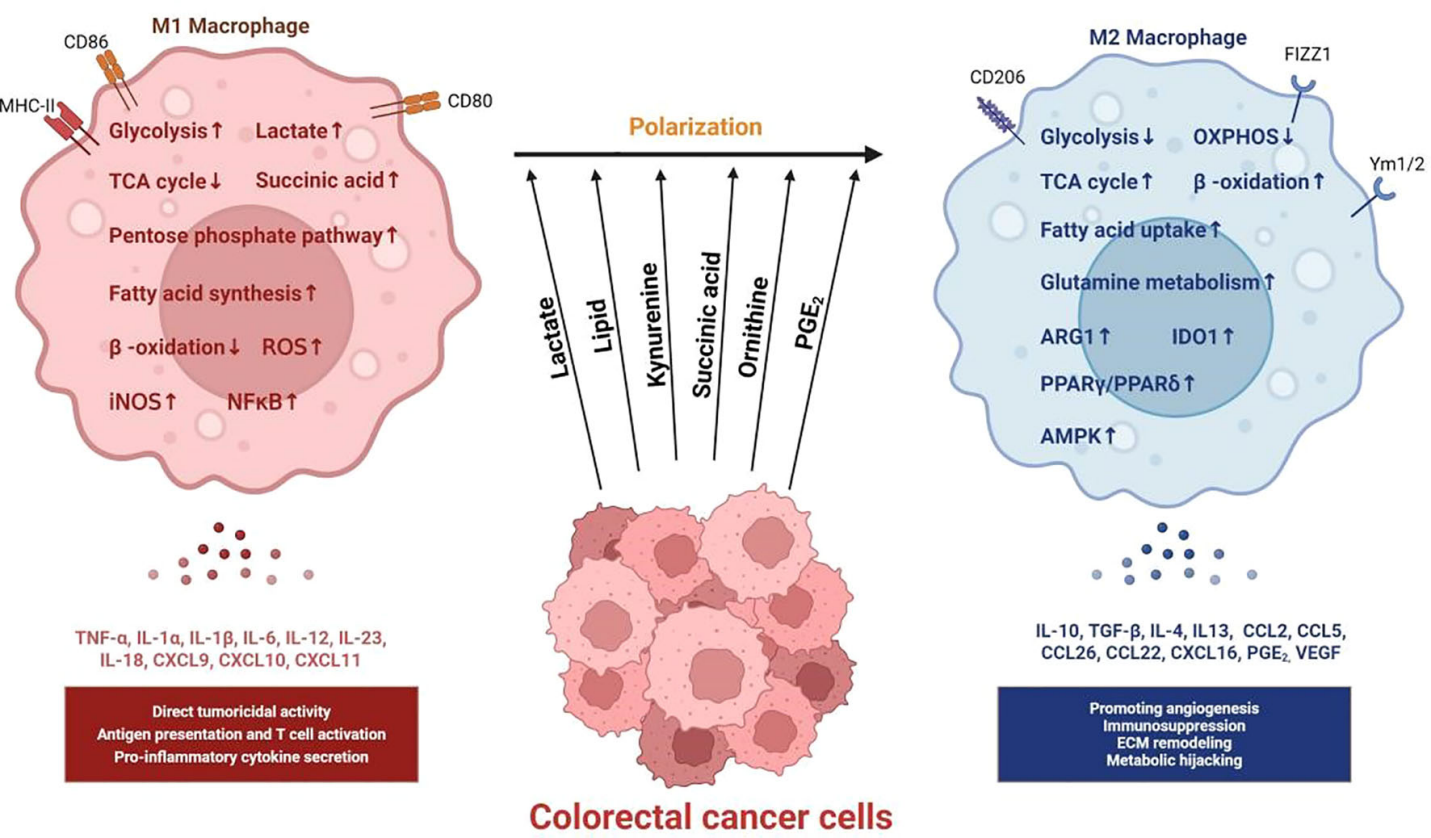
Macrophage polarization in the colorectal cancer microenvironment. Colorectal cancer cells secrete factors (lactate, lipid, kynurenine, succinic acid, ornithine, PGE_2_) promote the polarization of macrophages from an M1-like to an M2-like phenotype. M1-like macrophages show enhanced glycolysis, pentose phosphate pathway, fatty acid synthesis, and TCA cycle alteration, producing pro-inflammatory cytokines (TNF-α, IL-1β, etc.) for tumoricidal activity and T cell activation. M2 macrophages exhibit reduced glycolysis, enhanced OXPHOS, fatty acid β-oxidation, and glutamine metabolism, expressing markers (CD206, FIZZ1) and secreting cytokines (IL-10, TGF-β, etc.) that promote angiogenesis, immunosuppression, and metabolic hijacking. The polarization linked metabolic reprogramming shapes a tumor-favorable microenvironment in CRC (249).

Dendritic cells are the primary antigen-presenting cells in the body, playing a pivotal role in initiating adaptive immune cell activation. Activated DCs undergo metabolic reprogramming, marked by increased glycolysis and reduced OXPHOS, and enhanced glycolysis activates the STING pathway, thereby boosting bone marrow-derived DC-mediated anti-tumor responses (246, 247). Conversely, glucose deprivation in the TME suppresses glycolysis, impairing DC function. In CRC and melanoma, restoring glycolysis in tumor-infiltrating DCs rescues their functionality (248). Tumor-associated macrophages (TAMs) demonstrate polarization-dependent metabolism: proinflammatory M1-TAMs utilize glycolysis, while protumoral M2-TAMs favor OXPHOS/FAO (**Figure 5**) (249, 250). In the early stages of tumorigenesis, macrophages predominantly display the pro-inflammatory, anti-cancer M1 phenotype, which subsequently transitions to the anti-inflammatory, pro-cancer M2 phenotype as tumors progress. Glucose deprivation within the tumor microenvironment drives the polarization of M1 macrophages toward the immunosuppressive M2 phenotype.

Thus, CRC cells establish a glucose-deprived TME that: (i) directly starves effector T/NK cells of energy and biosynthetic precursors (a metabolic checkpoint), inducing dysfunction/exhaustion; (ii) selectively enriches immunosuppressive Tregs/MDSCs with superior metabolic fitness; and (iii) compromises antigen presentation—collectively dismantling antitumor immunity through immunometabolic sabotage.

### 5.2 Glutamine deprivation: impaired activation and function of antitumor immune cells

In the metabolically competitive tumor microenvironment of colorectal cancer, cancer cells exhibit voracious Gln consumption alongside glucose to meet critical demands for bioenergetics, redox homeostasis, and macromolecular biosynthesis. This results in significant Gln depletion, profoundly impacting immune cell functionality and shaping an immunosuppressive niche (**Figure 3**). Naive T cells primarily utilize FAO and OXPHOS with minimal Gln metabolism at rest, however, activated T cells induce ASCT2-mediated Gln uptake to support proliferation and effector cytokine (IL-2/IFN-γ) production (251). At this stage, T cells induce the expression of ASCT2, which mediates glutamine uptake and subsequently activates mTORC1 (252). Gln scarcity in the TME promotes Foxp3^+^ Treg differentiation in human CD4^+^ T cells by impairing nucleotide synthesis (253). Early effector T cells redirect glucose toward nucleotide biosynthesis while relying on Gln-derived anaplerosis to fuel TCA cycle-dependent ATP production and *de novo* pyrimidine synthesis (254).

In CRC or melanoma models, Gln supplementation enhances CD8^+^ T cell abundance and effector functions (IFN-γ/TNF/granzyme B) (255), whereas pharmacological inhibition or genetic ablation of tumor Gln metabolism (e.g., glutaminase knockout) not only suppresses cancer growth but also alleviates microenvironmental hypoxia, acidosis, and nutrient exhaustion, thereby augmenting CD8^+^ T cell antitumor activity (**Figure 4**) (256). Beyond supporting proliferation, Gln catabolism regulates T cell subset differentiation: Gls deficiency or glutaminase inhibition suppresses Th17 polarization by reducing Pik3ip1 expression while favoring Th1 responses (257). In contrast to effector T cells, Tregs and memory CD8^+^ T cells exhibit lower Gln dependence, preferentially utilizing OXPHOS/FAO (258). Consistently, Gln deprivation enhances Treg polarization and their immunosuppressive capacity against effector T cells (Teff) (259). Similarly, OXPHOS-dependent MDSCs undergo apoptosis upon Gln antagonism *in vitro (*260).

NK cells critically require Gln for proliferation and cytotoxicity, with Gln starvation markedly impairing their activity. In CRC liver metastases, myofibroblastic cancer-associated fibroblast (myCAF)-derived exosomal PWAR6 upregulates tumor cell SLC38A2 to deplete TME Gln, inducing NK cell dysfunction and apoptosis to accelerate metastatic progression (261). The functionality of tumor-associated macrophages is also impacted by Gln availability, M1-like TAMs depend on glucose and Gln for proinflammatory function (230). Whereas glutamine is critical for sustaining an active TCA cycle, M2 macrophages, which rely on oxidative phosphorylation for energy, are also dependent on glutamine metabolism. Studies have demonstrated that α-ketoglutarate (α-KG) derived from glutaminolysis promotes M2 activation via metabolic and epigenetic reprogramming (262). Gln deficiency also impairs the functional maturation of dendritic cells. Conventional dendritic cells (cDCs) comprise cDC1 and cDC2 subsets, which drive the priming of cytotoxic CD8^+^ T cells and CD4^+^ T cells, respectively. Moreover, cDC1 cross-presents tumor-associated antigens to cytotoxic CD8^+^ T cells, thereby augmenting anti-tumor immunity (263). In colorectal cancer or melanoma, glutamine supplementation enhances the maturation of intratumoral cDC1 and reinforces anti-tumor immunity. Conversely, specific knockout of the glutamine transporter SLC38A2 in cDC1 cells leads to reduced CD40 and MHC I expression, diminished IL-12 production, and impaired T cell priming capacity (255).

Thus, tumor-intrinsic Gln addiction creates a “metabolic immune checkpoint” in the TME, where competitive Gln depletion simultaneously starves effector immunity while enriching immunosuppressive populations—a dual mechanism of immunometabolic suppression in CRC.

### 5.3 Arginine starvation: a multi-source immunosuppressive niche

Arg, a conditionally essential amino acid, acts as a pivotal immunometabolic regulator in the colorectal cancer tumor microenvironment. Severe local depletion of arginine (“arginine starvation”) within this niche constitutes a fundamental mechanism of immune evasion (**Figure 3**). CRC cells exhibit marked arginine dependency to sustain rapid proliferation (via polyamine synthesis) and survival, aggressively competing for extracellular arginine through upregulated transporters (e.g. CAT1/SLC7A1). Arginine is indispensable for T cell proliferation and activation, for T cell expansion is completely abolished in arginine-free medium (264).

Arginine modulates T cell function through multiple pathways. Adequate arginine is critical for preserving the integrity and function of the CD3ζ chain, a key protein downstream of T cell receptor (TCR) signaling (265), whereas arginine restriction impairs TCR signal transduction. Additionally, arginine deficiency suppresses dephosphorylation of cofilin (an actin-binding protein), thereby compromising immune synapse formation and T cell activation (**Figure 4**) (266). Arginine is also essential for IL-2 signaling, as arginase deficiency downregulates IL-2 receptor α expression (267, 268). Moreover, arginase deficiency decreases the expression of several cytokines, including IFNγ and TNFα (266, 268). Exogenous arginine supplementation enhances T cell survival and antitumor immunity in murine models (264). Similarly, NK cell cytotoxicity and proliferation critically depend on arginine—depletion downregulates activating receptors (NKp46/NKp30), NKζ chain expression, and IFN-γ secretion (269). Perioperative arginine replenishment in CRC patients accelerates the recovery of NK cell cytotoxicity, interferon-γ secretion, and activating receptor expression to preoperative levels, while preventing postoperative metastasis (270).

Aside from tumor cell consumption, TAMs and MDSCs exacerbate arginine depletion via overexpression of arginase 1 (ARG1). In murine tumor models, high ARG1 expression is typically observed in monocyte-derived cells, whereas in humans, such high expression is most prevalent in polymorphonuclear cells rather than monocytes (271). Spatial analysis of tumors from colorectal cancer patients reveals that ARG1 expression in neutrophile granulocyte increases with proximity to the tumor (272). Numerous studies have established that elevated levels of ARG1-expressing MDSCs or overall upregulated ARG expression in tumors correlate with reduced T cell infiltration and poor prognosis (273). ARG1 expression is regulated by inflammatory cytokines: IL-4/IL-13 activate JAK-STAT6 signaling to induce ARG1 via STAT6-C/EBPβ/KLF4 complexes, while pro-inflammatory cytokines—including IL-6, tumor necrosis factor, interferon, Toll-like receptor (TLR) ligands, and PGE2—also upregulate Arg1 transcription via JAK1/JAK2 activation-induced recruitment and phosphorylation of STAT3, which then binds to specific regions of the Arg1 promoter in conjunction with C/EBPβ (273). Similar to ARG1, ARG2 exerts immunosuppressive effects by inducing extracellular arginine depletion. Though rarely reported, studies have shown that upregulated ARG2 expression in certain tumors, including acute myeloid leukemia (274), oral squamous cell carcinoma (275), and prostate cancer (276), contributes to immunosuppression. ARG2 is also highly expressed in immunosuppressive cells within the tumor microenvironment, such as DCs (277), Tregs (278), TAMs (277), and cancer-associated fibroblasts (276). Notably, both ARG2 deficiency or adoptive transfer of ARG2-deficient CD8^+^ T cells enhance CD8^+^ T cell activation and effector functions, thereby impairing the growth of B16 and MC38 tumors.

Immunosuppressive ARG1 activity competitively inhibits NOS2-mediated nitric oxide (NO) production (279). Typically, M1-TAMs exhibit high NOS2/low ARG1, whereas M2-TAMs show low NOS2/high ARG1 (**Figure 5**). In CRC, NOS2 expression correlates with favorable prognosis and enhanced CD8^+^/CD4^+^ T cell infiltration, with NOS2 being essential for the efficacy of adoptive CD8^+^ T cell therapy (280). Substrate supplementation-induced endogenous nitric oxide (NO) production in intestinal epithelial cells improves epithelial integrity and mitigates colitis and inflammation-associated colon cancer (228). A direct consequence of NOS2 downregulation and ARG upregulation is elevated ornithine levels in the microenvironment, and L-ornithine, a precursor for polyamine and proline synthesis with immunomodulatory properties, accumulates to impair T cell function (273). However, in colorectal cancer, ornithine treatment enhances T cell function by reducing ammonia levels, which would otherwise induce T cell exhaustion, while also potentiating the efficacy of anti-PD-L1 immunotherapy in CRC (281).

Ornithine is converted into polyamines (putrescine, spermidine, and spermine) via ornithine decarboxylase (ODC). In macrophages, ornithine decarboxylase exacerbates colitis and promotes colitis-associated colon carcinogenesis by inhibiting M1 polarization (282). Additionally, polyamines regulate the functions of various immune cells: they suppress T cell responses by upregulating IL-10 expression and downregulating IFNγ and IL-2 levels; in NKT cells, polyamines enhance the release of IL-4 and IL-13 while inhibiting the expression of perforin, IL-12, and IFNγ; furthermore, polyamines drive M2-like polarization and upregulate the expression of ARG1, IL-4, and IL-13 (273).

Collectively, the CRC TME establishes “arginine starvation” through tumor-intrinsic uptake and MDSC/TAM-mediated ARG1 catabolism, creating an immunosuppressive niche that simultaneously impairs effector immunity and enriches regulatory populations.

### 5.4 Tryptophan depletion and kynurenine accumulation: an immunosuppressive axis

The profound depletion of tryptophan coupled with significant accumulation of its catabolite kynurenine (Kyn) forms a potent immunosuppressive axis in the colorectal cancer tumor microenvironment, thus remodeling immune cell function and facilitates tumor immune evasion (**Figure 3**). As an essential amino acid, Trp serves not only as a protein building block but also as the substrate for the kynurenine pathway—a critical immunoregulatory cascade. In the CRC TME, malignant cells, TAMs, MDSCs inhibitory DCs, and cancer-associated fibroblasts markedly overexpress indoleamine 2,3-dioxygenase (IDO), the rate-limiting enzyme of Trp catabolism (283, 284). Elevated IDO expression in CRC patients correlates significantly with reduced overall survival and diminished immune cell infiltration (197). Meanwhile high IDO expression colorectal tumor enable partial tumor subsets to suppress the infiltration of CD3^+^ T cells, CD8^+^ T cells, and CD57^+^ NK cells in CRC via local tryptophan depletion and production of pro-apoptotic tryptophan catabolites, thus evading immune attack (285). Tumor-derived IDO further orchestrates local and systemic immunosuppression by recruiting and activating MDSCs and Tregs, while IDO inhibition reduces these populations and attenuates their suppressive functions (286).

Trp depletion activates the amino acid starvation response (GCN2 kinase pathway) in CD8^+^ T cells, inducing cell cycle arrest at the G1/S phase and promoting apoptosis, whereas Kyn supplementation directly suppresses T cell proliferation (**Figure 4**) (287). Kyn accumulation and its signaling through the aryl hydrocarbon receptor (AhR)—widely expressed in cancer cells and immune cells (T cells, macrophages, Tregs, DCs, MDSCs)—drives important immunosuppressive mechanisms (288). In T cells, kynurenine induces high PD-1 expression by activating the AhR receptor, promoting the exhaustion of tumor-infiltrating T cells and thereby enabling evasion of immune killing (289). Moreover, kynurenine competes with other amino acids, such as methionine and leucine, that are transported into T cells via Slc7a5, inhibiting amino acid uptake by T cells and thus impairing T cell-mediated immune responses (290). In APC-mutant CRC, Kyn-AhR activation enhances glycolysis and CXCL5 secretion to recruit macrophages (200). The Kyn-AhR axis additionally polarizes macrophages toward M2 phenotypes and expands Treg populations (291), while AhR blockade in AOM/DSS models reverses TAM polarization, suppresses Tregs, and enhances CD8^+^ T cell infiltration to ameliorate colitis-associated carcinogenesis (292).

Furthermore, in colorectal cancer, intestinal enterochromaffin (EC) cells and certain immune cells convert a portion of tryptophan (Trp) to 5-hydroxytryptamine (5-HT) via tryptophan hydroxylase 1 (TPH1), collectively exacerbating Trp starvation in the tumor microenvironment. Notably, 5-HT itself functions as a key immunomodulatory molecule: in the intestine, 5-HT secreted by EC cells tends to exert pro-inflammatory effects, in contrast to the anti-inflammatory properties of neuron-synthesized 5-HT (293). In Tph1-knockout mice, reduced epithelial 5-HT synthesis alleviates dextran sulfate sodium (DSS)-induced intestinal inflammation (294); conversely, DSS-induced inflammation is exacerbated in Tph2-knockout mice (293). During intestinal inflammation, 5-HT activates dendritic cells to promote IL-12 production, while 5-HT signaling further induces NF-κB activation in these cells (295). This enables dendritic cells to prime CD4^+^ T cells, which in turn secrete IL-17 and interferon (IFN)-γ, thereby aggravating colitis. Additionally, 5-HT regulates macrophage infiltration in DSS-induced colitis and stimulates pro-inflammatory cytokine production, exacerbating colonic inflammatory damage (296).

In CRC patients, serotonin concentrations in metastatic lesions correlate negatively with the number of CD8^+^ tumor-infiltrating T cells. Serotonin depletion reduces CRC tumor growth, increases CD8^+^ T cell infiltration, and downregulates PD-L1 expression via serotonylation (297). The blockade of serotonin transporter (SERT), which regulates serotonin levels by mediating its uptake from the extracellular to intracellular environment, enhances cytotoxic CD8^+^ T cell anti-tumor responses and inhibits tumor growth in multiple mouse models, including CRC models (298). In cancer-associated fibroblasts (CAFs), 5-HT promotes serotonylation of histone H3 at glutamine 5 (H3Q5ser), this modification triggers the transition of CAFs to an inflammatory CAF (iCAF) subtype, thereby enhancing CRC proliferation, invasion, and macrophage polarization (299).

Thus, in the CRC TME, three factors synergize to form an extensive immunosuppressive network: Trp depletion driven by excessive IDO1/TDO2 activity, massive kynurenine (Kyn) accumulation, and elevated 5-HT levels resulting from Trp shunting into 5-HT synthesis. This intertwined Trp metabolic disorder plays a central role in CRC progression, metastasis, and resistance to immunotherapy.

### 5.5 Lactate accumulation: acidification and inhibition of antitumor immunity

In the complex, dynamic tumor microenvironment of colorectal cancer, metabolic reprogramming of cancer cells—particularly the enhanced Warburg effect—serves not only as a core strategy to meet bioenergetic and biosynthetic demands but also as a key driver of immunosuppression. Lactate, the end product of this aberrant glycolysis, emerges as a critical immunomodulatory effector in the CRC TME (**Figure 3**). CRC cells overexpress glucose transporters (e.g., GLUT1) to facilitate massive glucose uptake, preferentially undergoing glycolysis over oxidative phosphorylation even under normoxia, resulting in substantial lactate production. Lactate is then efficiently extruded via monocarboxylate transporters (primarily MCT4), a proton-coupled symporter that co-exports H^+^ with lactate (43). The resultant lactate accumulation, combined with H^+^ extrusion by tumor cell H^+^-ATPases and Na^+^/H^+^ exchangers (NHEs), directly acidifies the TME (extracellular pH [pHe] ≈6.5–7.0 vs. physiological 7.4) while maintaining a slightly alkaline intracellular pH (pHi >7.4) (300).

This lactate-driven acidosis profoundly suppresses immune cell function, constituting a major immune evasion mechanism. For cytotoxic T lymphocytes (CTLs) and NK cells, lactate-induced intracellular acidification impairs nuclear factor of activated T cells (NFAT) signaling, reducing production of effector cytokines (IFN-γ, TNF-α, granzyme B) (**Figure 4**) (301). In colorectal cancer, KRAS-mutant tumors exhibit greater lactate production than wild-type tumors, and elevated lactate levels resulting from KRAS mutations sensitize tumor-specific cytotoxic CD8^+^ T cells to activation-induced cell death (AICD) by suppressing NF-κB activity, thereby reducing their infiltration into the tumor microenvironment (302). Diminished infiltration of cytotoxic CD8^+^ T cells also lowers the sensitivity of KRAS-mutant colorectal cancer to anti-PD-1 therapy. Pharmacological MCT inhibition reduces lactate efflux in CRC, enhancing T cell infiltration and checkpoint blockade efficacy (303). In CRC liver metastases, TME acidosis triggers mitochondrial dysfunction in liver-resident NK cells, inducing apoptosis (304). Lactate dehydrogenase inhibition (LDHi) reduces tumor glucose uptake, thereby augmenting T cell glycolysis and antitumor immunity (240).

Unlike Teff cells, Tregs thrive in high-lactate conditions: lactate promotes MOESIN lactylation and TGF-β signaling to enhance Treg differentiation and immunosuppressive function (305). In CRC, lactate upregulates CTLA-4 via Foxp3-dependent USP39-mediated RNA splicing (306), while MCT1-mediated lactate uptake facilitates NFAT1 nuclear translocation to boost PD-1 expression (307). MDSCs are heavily infiltrated in the colorectal cancer tumor microenvironment, with lactate acting as a key driver of their expansion and immunosuppressive functions. Lactate promotes MDSC expansion, recruitment, and immunosuppressive activity via pathways such as GPR81/mTOR/HIF-1α/STAT3, thereby suppressing T cell function (308). Additionally, activation of the lactate receptor HCAR1 signaling pathway in colorectal tumor cells induces expression of chemokines CCL2 and CCL7, which in turn recruits immunosuppressive CCR2+ polymorphonuclear myeloid-derived suppressor cells (PMN-MDSCs) into the TME (309).

For TAMs, lactate serves as a potent polarizing signal. A high-lactate environment drives the polarization of macrophages toward the pro-tumor, pro-angiogenic, and immunosuppressive M2 phenotype by activating signaling pathways such as Gpr132, Olfr78, HIF-1α, Nrf2, and STAT3 (310–314). These M2-type TAMs secrete factors including transforming growth factor-β (TGF-β), interleukin-10 (IL-10), arginase-1 (ARG1), and vascular endothelial growth factor (VEGF), which further suppress anti-tumor immunity and promote angiogenesis (**Figure 5**). Additionally, lactate produced by colorectal cancer can induce M2 macrophage polarization by activating the AKT-ERK pathway; following polarization, M2 macrophages secrete abundant CCL8, which accelerates tumor progression and metastasis via the CCR5/mTORC1 pathway (315). In CRC, Lactate derived from cancer cells induces the cytoplasmic-to-nuclear translocation of the transcription factor Ap-2α in TAMs. Subsequently, Ap-2α activates the expression of Sirpα in macrophages via Elk-1, thereby inhibiting macrophage phagocytosis (308). Furthermore, lactate imposes a tolerogenic phenotype on DCs, impairing antigen presentation and IFN-α/IFN-γ production to weaken T cell priming (309). Notably, in colorectal cancer, as metabolic products of the intestinal microbiota, lactate and its derivatives can also modulate immune cell function (316).

Thus, CRC cells construct a multifaceted immunosuppressive network through lactate overproduction: acidosis directly cripples effector lymphocytes while bolstering immunosuppressive populations (M2-TAMs, MDSCs, Tregs) and sabotaging antigen presentation.

### 5.6 Accumulation of TCA cycle intermediates: immune-modulatory effects

Besides glycolytic reprogramming and lactate secretion, aberrant TCA cycle activity and pathological accumulation of key intermediates constitute equally pivotal mechanisms shaping the immunosuppressive tumor microenvironment in colorectal cancer. Compared to lactate’s broad suppressive effects, these metabolites exhibit greater cell-type and metabolite specificity. CRC cells frequently remodel their TCA cycle due to mutations (KRAS, TP53), hypoxia, or dysregulated signaling, leading to intracellular accumulation and extracellular release of specific intermediates—including succinate, fumarate, itaconate, and α-ketoglutarate (α-KG)—which function as potent immunometabolites by directly modulating immune cell metabolism, epigenetic programming, and signaling pathways (**Figure 3**).

In general, the succinate-SUCNR1 axis acts as a potent pro-inflammatory signal: SUCNR1 is highly expressed in DCs, where succinate binding activates ERK1/2 to enhance production of inflammatory mediators and T cell priming (317). In murine colitis models, exogenous succinate promotes FOXP3 degradation to impair Treg-mediated immunosuppression, exacerbating inflammation (318). Autocrine/paracrine succinate also boosts macrophage IL-1β secretion (319). Paradoxically, in the TME, tumor-derived succinate becomes protumorigenic, CRC-secreted succinate recruits macrophages via SUCNR1 and drives TAM polarization through PI3K/AKT and HIF-1α activation (**Figure 5**) (320). While High concentrations of succinate in the tumor microenvironment are taken up by T cells, suppressing glucose flux through the TCA cycle, this, in conjunction with SUCNR1 signaling, inhibits T cell functions (321).

Fumarate accumulates significantly in colorectal cancers harboring fumarate hydratase (FH) mutations, while in sporadic CRC, it also accumulates due to TCA cycle blockage. Fumarate released by tumors targets ZAP70—a key kinase in the T cell receptor (TCR) signaling pathway—via succinylation, its inactivation blocks TCR signal transduction, preventing CD8^+^ T cells from effective activation, proliferation, and secretion of effector factors (e.g., IFN-γ, TNF-α), thereby impairing their tumor-killing capacity (322).

α-KG is a key intermediate in the TCA cycle and an important cofactor for dioxygenases, including TET and JMJD histone demethylases. αKG exerts a significant impact on the activation of macrophages: it promotes M2 macrophage polarization via JMJD3-dependent epigenetic reprogramming while suppressing M1 proinflammatory responses by inhibiting NF-κB through PHD-mediated IKKβ hydroxylation (262). Reports have demonstrated that α-KG coordinates the M1/M2 phenotypic balance of alveolar macrophages via immune response modulators (PPARγ or mTORC1), thereby impacting sepsis-associated ALI/ARDS (323). While the effect of αKG accumulation on immunity in colorectal cancer remains unreported, in cholangiocarcinoma, α-KG accumulation activates the OXGR1 receptor on macrophages, which in turn triggers the MAPK signaling pathway, suppresses macrophage antigen presentation, and enhances immunosuppression (324). In colitis models, increased αKG/succinate ratio resulting from αKG accumulation can enhance the activity of αKG-dependent dioxygenases, thereby promoting the differentiation of intestinal stem cells and maintaining intestinal homeostasis, αKG supplementation can reverse these colitis-induced injuries and facilitate tissue healing (325). Notably, when IDH1/2 is mutated, the enzyme catalyzes α-KG to generate the oncogenic metabolite D-2-hydroxyglutarate (D-2HG), despite the very low frequency of IDH mutations in colorectal cancer, studies have demonstrated that D-2HG administration during colitis delays colitis resolution and accelerates tumorigenesis (326).

Itaconate is primarily produced by myeloid immune cells, macrophages in particular, via the decarboxylation of cis-aconitate, a reaction catalyzed by ACOD1, which is activated upon stimulation by various inflammatory factors. It exerts anti-inflammatory effects through multiple mechanisms, including inhibiting succinate dehydrogenase activity, activating Nrf2 via KEAP1, and competitively inhibiting TET2 with α-KG to regulate inflammatory factor expression (327). In colorectal cancer, macrophages serve as the main source of itaconate; knockout of ACOD1 in myeloid cells significantly suppresses the malignant proliferation of multiple tumors, including colorectal cancer (328). Within the tumor microenvironment, tumor cells take up itaconate via the itaconate transporter SLC13A3, thereby evading immune-mediated ferroptosis through activation of the Nrf2-SLC7A11 pathway (328). Additionally, itaconate inhibits CD8^+^ T cell proliferation and their anti-tumor immune function by suppressing aspartate/serine synthesis (329).

Thus, CRC cells transform TCA cycle intermediates from mere energy currencies into sophisticated immunoregulatory signals through pathological accumulation and secretion, creating a metabolite-specific immunosuppressive landscape that complements broader lactate-mediated suppression.

### 5.7 Lipid accumulation: metabolic reprogramming and immune suppression

Metabolic reprogramming in colorectal cancer profoundly alters lipid metabolism, transforming lipid species from energy storage molecules into potent immunoregulatory signals that actively shape the immunosuppressive tumor microenvironment through multifaceted mechanisms (**Figure 3**). CRC cells enhance *de novo* fatty acid synthesis via upregulation of FASN, SCD1, and ACC, while increasing exogenous free fatty acid (FFA) uptake through overexpression of LDL receptor (LDLR), CD36, and fatty acid transport proteins (FATPs) to meet demands for membrane biosynthesis, signaling, and energy storage. Excess fatty acids are either stored as triglycerides in lipid droplets or metabolized through β-oxidation, phospholipid synthesis, or conversion to signaling lipids (e.g., arachidonic acid derivatives) and cholesteryl esters (CEs). These lipid species are actively secreted (via exosomes or free diffusion) or passively released through necrosis, creating a lipid-rich TME that directly modulates immune cell fate and function.

TAMs and Tregs in the tumor microenvironment depend on FAO for energy, accumulation of fatty acids in this microenvironment promotes their survival and drives pro-tumor effects (330). Unsaturated fatty acids (USFAs)—but not saturated fatty acids (SFAs)—induce intracellular lipid droplet (LD) formation and suppress the activity of MDSCs (331). CD8^+^ T cells cannot catabolize accumulated intercellular very-long-chain fatty acids (FFAs) nor store them in lipid peroxides, a lipid-rich TME thereby triggers severe lipotoxicity and subsequent T cell exhaustion (332).

In various tumors, including melanoma, colon cancer, and breast cancer, elevated cholesterol levels in the TME impair the anti-tumor activity of CD8^+^ T cells and induce their exhaustion (**Figure 4**) (333). Concurrently, cholesterol released by tumor cells into the TME drives immunosuppressive reprogramming by activating myeloid-derived suppressor cells (334). Cholesterol enriched in the tumor microenvironment induces immune checkpoint expression and CD8^+^ T cell exhaustion by increasing endoplasmic reticulum stress in CD8^+^ T cells (333). Additionally, cholesterol stimulates TAMs to secrete CCL5, which stabilizes PD-L1 via p65/STAT3-CSN5 signaling to facilitate immune escape. Intermediates in the cholesterol synthesis process also play a role in regulating immune function (335). Activation of the SREBF2 gene in CRC cells promotes the accumulation of cholesterol biosynthesis intermediates and their release into the tumor microenvironment. Among these, lanosterol is taken up by CD8^+^ T cells, where it inhibits the mevalonate pathway and reduces KRAS isoprenylation. And diminished KRAS isoprenylation suppresses downstream KRAS signaling, ultimately leading to CD8^+^ T cell inactivation (336). Squalene secreted by tumors can modulate the NF-κB pathway through p65, represses CXCL1 transcription, and thereby reduces the recruitment of MDSCs and TAMs into the tumor microenvironment (337).

Cholesterol is highly prone to oxidation, generating a series of oxidized cholesterol species. In CRC cells, cholesterol uptake causes endoplasmic reticulum cholesterol accumulation and increased 24-hydroxycholesterol (24-HC) production, thus upregulating TGF-β1 via LXR signaling activation. And TGF-β1, released into the TME, activates the SMAD3 pathway in memory CD8^+^ T cells, ultimately impairing their anti-tumor activity (338). 25-hydroxycholesterol (25-HC) secreted by colorectal cancer cells inhibits the phagocytic activity of cytotoxic lymphocytes (CTLs), thereby suppressing anti-tumor immunity (339). 27-hydroxycholesterol (27-HC) alters the LXR and SREBP2 pathways, depleting cholesterol reserves and inducing T cell exhaustion and dysfunction (340). 7-dehydrocholesterol (7-DHC) regulates the production of type I interferons (IFNs) by modulating AKT3 activation (341). In addition, oxidized cholesterol generated by colorectal cancer cells inhibits CCR7 expression on dendritic cells and dampens anti-tumor responses via activation of liver X receptor (LXR) signaling (342). Notably, cholesterol is a precursor of steroid hormones, although colorectal cells are not the primary synthesis organs, locally concentrated steroids may be involved in regulating immune homeostasis.

Prostaglandin E2 (PGE^2^)—a major metabolite derived from arachidonic acid (AA) via the cyclooxygenase-2 (COX-2) and microsomal prostaglandin E synthase-1 (mPGES-1) pathway—is ubiquitously overexpressed in colorectal cancer and functions as a key immunosuppressive mediator. The homeostatic accumulation of PGE2 in intestinal tumors arises from enhanced COX-2-driven biosynthesis and reduced degradation due to the pervasive loss of 15-hydroxyprostaglandin dehydrogenase (15-PGDH) in CRC (343). PGE_2_ primarily regulates immune cells via EP2 and EP4 receptors, the dominant receptors on immune cells. The COX-2–PGE_2_ pathway drives tumor immune evasion in intestinal cancer by modulating immunosuppressive cells (MDSCs, Tregs, macrophages), antigen-presenting cells (APCs: dendritic cells and macrophages), and lymphocytes (CD4^+^ T cells, CD8^+^ T cells, NK cells). *In vitro* PGE_2_ promotes M2-like macrophage polarization in bone marrow-derived macrophages via the CREB/CRTC pathway (**Figure 5**) (344), and *in vivo* PGE_2_ inhibits CD8^+^ T cells by inducing PD-L1 expression in TAMs (345). PGE_2_ directly suppresses CD8^+^ T cell proliferation, cytotoxic activity (with reduced granzyme/perforin expression), and effector cytokine production (e.g., IFN-γ, TNF-α). PGE_2_ can also promote the differentiation and migration of Tregs (346), and induce the accumulation of tumor-associated MDSCs to facilitate colorectal cancer growth (347). Additionally, PGE_2_ inhibits dendritic cell differentiation and maturation (348), impairing their antigen-presenting capacity, and suppresses NK cell function (349), thereby forming a multi-layered immunosuppressive network.

Thus, CRC cells remodel lipid metabolism to create a TME enriched with immunoregulatory lipids that simultaneously cripple effector immunity while bolstering immunosuppressive populations, forging a lipid-driven immunosuppressive niche.

### 5.8 The gut microbiota-metabolism-immunity axis in colorectal cancer

The tumor microenvironment of colorectal cancer is deeply integrated into the complex intestinal ecosystem, where gut microbiota and their diverse metabolic activities act as intimate neighbors and dynamic metabolic factories for the tumor. Gut dysbiosis is a hallmark of CRC, with microbiota driving carcinogenesis through multiple mechanisms: genotoxic effects of specific pathogens (e.g., colibactin produced by pks^+^*Escherichia coli* inducing DNA damage) (350); induction of inflammation (e.g., Bacteroides fragilis-triggered, IL-17-dominated mucosal inflammation promoting epithelial mutations) (351); facilitation of immune evasion (e.g., *Fusobacterium nucleatum* suppressing antitumor immunity via adhesins Fap2 and CbpF) (352, 353); and activation of oncogenic pathways (e.g., B. fragilis toxin BFT activating Wnt/β-catenin signaling (354), *F. nucleatum* inducing Wnt/β-catenin via Annexin A1 (355)).

Additionally, microbial metabolites, including secondary bile acids (e.g., deoxycholic acid) and short-chain fatty acids (SCFAs, e.g., butyrate), further regulate tumor progression through epigenetic modulation and inflammatory responses (356). Microbial metabolites serve as core mediators of microbiota-host immune crosstalk. SCFAs, including acetate, butyrate, and propionate, are primarily generated through the fermentation of dietary fiber in the gut by specific anaerobic bacterial subgroups, notably members of the genera Clostridium, Eubacterium, and Butyrivibrio. Clostridium clusters IV and XIVa are highly efficient butyrate producers. Within the human colon, the dominant species of Clostridium cluster IV is *Faecalibacterium prausnitzii*, whereas Roseburia spp., Anaerostipes, and Eubacterium represent key butyrate-producing constituents of Clostridium cluster XIVa (357). Most SCFAs serve as an energy source for colonic cells, with butyrate being the primary one, and it is estimated that 60% to 70% of the energy supply for normal colonic cells is derived from SCFA oxidation (358). Short-chain fatty acids unutilized by colonic cells, primarily acetate and propionate, enter the portal circulation and are transported to the liver (359).

SCFAs inhibit histone deacetylases (HDACs); specifically, butyrate inactivates NF-κB and downregulates proinflammatory cytokine expression in monocytes, neutrophils, and macrophages (360). Binding of SCFAs to FFAR2 and GPR109a on intestinal epithelial cells activates NLRP3 inflammasomes and induces IL-18 release, maintaining intestinal integrity and homeostasis (361). In dendritic cells, butyrate and propionate suppress proinflammatory cytokine release and inhibit LPS-induced IL-6/IL-12 expression (362). SCFAs promote Treg differentiation via HDAC9 inhibition-mediated FOXP3 upregulation and enhance FOXP3^+^ Treg function to suppress colitis (363). As ligands for G protein-coupled receptors (GPCRs), SCFAs activate GPR41, GPR43, and GPR109A to modulate immunity: GPR43 activation induces NLRP3 inflammasome assembly, epithelial IL-18 secretion, neutrophil recruitment, and Treg differentiation/suppression (364); GPR109A activation increases monocyte anti-inflammatory mediators and induces Tregs to prevent colitis and carcinogenesis (365). CRC patients exhibit reduced butyrate-producing Clostridiales; notably, Clostridiales supplementation in murine CRC models elevates butyrate levels, increasing intratumoral IFN-γ^+^ CD8^+^ T cells and NK cells to enhance antitumor immunity (366).

Secondary bile acids (BAs)—including deoxycholic acid (DCA), lithocholic acid (LCA), and ursodeoxycholic acid (UDCA)—are derived from primary BAs (cholic/chenodeoxycholic acids) via gut microbial metabolism. Elevated colonic secondary BAs in high-fat diet cohorts correlate with increased CRC risk (367), promoting tumorigenesis through epithelial barrier disruption, oxidative DNA damage, and NF-κB activation (368). Derivatives of LCA/DCA (iso-, 3-oxo-LCA/DCA, allo-, 3-oxoallo-, isoalloLCA) regulate TH17/Treg differentiation, among them 3-oxoLCA binds RORγt to inhibit TH17 cells, while isoalloLCA enhances FOXP3^+^ Tregs via mitochondrial ROS (369, 370). In addition, isoDCA promotes Treg differentiation through FXR-mediated dendritic cell suppression (371). DCA accumulates in high-fat diet-induced colitis models, polarizing macrophages toward M1 phenotypes via TLR2 to increase proinflammatory cytokines (372). In CRC patients, DCA accumulation impairs CD8^+^ T cell effector function by inhibiting Ca²^+^-NFAT2 signaling, accelerating tumor growth (373).

Gut microbiota also intersect with amino acid metabolism: certain microbes deplete Trp for catabolism or convert it to kynurenine, exacerbating Trp starvation and AhR-mediated immunosuppression while suppressing indole derivative production. Microbially derived indoles (indole, indole-3-acetic acid/IAA, indole-3-propionic acid/IPA, indole-3-lactic acid/ILA) critically influence CRC progression: ILA—the most extensively studied anticancer indole—activates CD8^+^ T cells by enhancing dendritic cell IL-12 production via histone modification and chromatin accessibility (316), promotes effector molecule release by inhibiting the cholesterol metabolism gene Saa3 in CD8^+^ T cells (316), and reduces CD86^+^ proinflammatory macrophages via AhR/p-AKT/IL-1β signaling (374). High-fiber/anthocyanin-rich black rice diets increase probiotics and ILA levels to inhibit CRC (375). IPA enhances H3K27 acetylation at the Tcf7 super-enhancer, driving CD8^+^ T cell differentiation toward Tpex cells and improving immune checkpoint inhibitor efficacy in melanoma, breast cancer, and CRC (376). IAA suppresses tumorigenesis by inducing IL-35 production in macrophages, T cells, and B cells to alleviate precancerous inflammation (377).

Altered microbial arginine metabolism accelerates CRC in AOM/DSS models: reduced bacterial arginine catabolism elevates TME arginine levels, activating NO synthesis, polyamine production, angiogenesis, M2 macrophage polarization, and Wnt/β-catenin signaling (377).

The microbiota-metabolism-immunity axis constitutes a central hub in CRC pathogenesis, offering unique therapeutic targets—including probiotics/prebiotics/synbiotics, dietary interventions (e.g., high-fiber diets to boost SCFAs), metabolite antagonists (e.g., AhR inhibitors), precision antibiotics, *F. nucleatum* antibodies, and promising fecal microbiota transplantation (FMT)—to restore healthy microbial composition/function, reverse immunosuppression, and enhance antitumor immunotherapy (e.g., immune checkpoint blockade).

### 5.9 Metabolism intervention and immunotherapy in colorectal cancer

Immunotherapy for colorectal cancer, particularly immune checkpoint blockade, has achieved substantial success in CRC patients with dMMR/MSI-H. However, it exhibits limited efficacy in the predominant subset of patients with pMMR/MSS, highlighting the urgent need to mitigate immunosuppression within the tumor microenvironment (378). Targeting reprogrammed metabolic pathways in CRC to reverse immunosuppression and reshape the immune microenvironment, thereby boosting the efficacy of existing immunotherapies, has emerged as a highly promising therapeutic approach.

The core goal of metabolic intervention is to eliminate metabolic barriers in the TME, establishing favorable conditions for the survival and functional performance of effector immune cells (particularly CD8^+^ T cells) while concurrently attenuating the activity of immunosuppressive populations (e.g., Tregs and MDSCs). Tumor cells outcompete immune cells for key nutrients (e.g., glucose and glutamine) via the Warburg effect and glutamine addiction. This leads to drastically reduced proliferative capacity and cytotoxicity in tumor-infiltrating CD8^+^ T cells and NK cells due to insufficient metabolic substrates, rendering them poorly responsive to immune checkpoint blockade. Knocking down LDHA to reduce tumor cell glycolytic activity increases glucose availability in the TME, thereby maximizing Treg destabilization and subsequently enhancing the therapeutic efficacy of CTLA-4 blockade (379). Similarly, treatment with lactate dehydrogenase inhibitors (LDHi) reduces tumor cell glucose uptake and expression of the glucose transporter GLUT1, improving the tumor-killing function of T cells and dampening Treg immunosuppressive activity. Combining LDHi with PD-1 and CTLA-4 antibodies further enhances the efficacy of CRC immunotherapy (240). An acidic microenvironment is a hallmark of the TME. The Warburg effect in tumors drives substantial lactate accumulation, which not only directly inhibits CD8^+^ T cell activation and function but also promotes M2 macrophage polarization and Treg expansion, further exacerbating immunosuppression. Tumor-derived lactate also impairs the interaction between PD-L1 and anti-PD-L1 antibodies, contributing to ICB resistance, however, combining the monocarboxylate transporter 1 (MCT1) inhibitor AZD3965 with anti-PD-L1 antibodies effectively reduces lactate efflux and treats tumors refractory to PD-1/PD-L1 blockade (380).

Glutamine is a critical energy source for tumor cells. Its metabolic reprogramming not only sustains tumor proliferation but also restricts T cell metabolic adaptability and function by depleting microenvironmental glutamine. Glutamine deprivation can inhibit tumor growth, however, it can also upregulate PD-L1 expression in various cancer cells, including CRC cells, enhancing immune escape (381). Additionally, it promotes autophagy-dependent degradation of the IFN-γ receptor (IFNGR1), conferring resistance to anti-PD-1/PD-L1 therapy (382). Thus, targeting glutamine utilization as a standalone therapeutic strategy often proves insufficient to achieve durable anti-tumor effects, in contrast, combining glutamine metabolism inhibition with ICB effectively reverses such adaptive resistance and augments the magnitude and persistence of anti-tumor immune responses (381, 382). Arginine serves as an indispensable nutrient for T cell proliferation and activation, as it supports critical metabolic processes and signaling pathways that drive effector T cell expansion and functional maturation. Notably, inhibition of ARG1 effectively relieves the metabolic constraint on T cells and promotes their robust expansion. Consistent with this, inhibition of ARG1 can significantly enhances the therapeutic efficacy of anti-PD-L1 immunotherapy by reversing the arginine-depleted TME (383). Kynurenine can induce Treg differentiation, upregulate PD-1 on CD8^+^ T cells, and accelerate intestinal carcinogenesis. IDO1 inhibitor monotherapy reduces intratumoral kynurenine levels by over 80% and inhibits tumor growth, while combination with anti-PD-L1 antibodies further enhances efficacy (384). Thus far, various IDO1-targeted inhibitors have shown promise in Phase I/II trials, but successes have not translated to Phase III. This does not diminish the importance of kynurenine limitation, instead, it highlights the need for precise patient stratification, optimized combination strategies, targeting additional pathway nodes, or directly inhibiting AhR with antagonists. For the 5-hydroxytryptamine pathway, exploring selective 5-HT receptor antagonists or inhibiting TPH1 to reduce intratumoral 5-HT production represents a promising direction. Ketanserin, a selective 5-HT2A receptor inhibitor, reverses the suppressed cytotoxic phenotype of CD8^+^ T cells, and combining it with anti-PD-L1/anti-VEGFA antibodies significantly prolongs mouse survival (385). Blocking the serotonin transporter (SERT), which regulates 5-HT levels via intracellular uptake, can also enhance cytotoxic CD8^+^ T cell responses and exhibits synergistic effects with PD-1 blockade (298).

Regarding fatty acid metabolism, FASN and CPT1A are promising targets. FASN regulates *de novo* fatty acid synthesis, two distinct inhibitors (orlistat and TVB-2640) can strongly inhibited tumor growth combined with anti-PD-L1 antibodies (386). CPT1A controls fatty acid flux into mitochondria for β-oxidation, the inhibitor etomoxir (ETO) slightly reduced CRC growth alone but significantly extended survival when combined with anti-PD-L1 antibodies (387). ACLY, a key cytosolic enzyme that converts mitochondrial citrate to acetyl-CoA, exerts dual effects when inhibited, it not only activates the cGAS-STING innate immune pathway but also concomitantly upregulates PD-L1 expression in cancer cells. Combining ACLY inhibition with anti-PD-L1 antibodies enhances immunotherapeutic efficacy and reverses the resistance to anti-PD-L1 antibodies in a cGAS-STING-dependent manner (388).

High-fiber diets are intended to boost the production of SCFAs, particularly butyrate, via fermentation by beneficial gut bacteria. Butyrate supplementation effectively inhibits the growth of orthotopic CRC tumors, however, it fails to enhance the efficacy of anti-PD-1 antibodies in MSI-High tumors. In contrast, it significantly improves anti-PD-1 responses in PD-1 refractory microsatellite stable CRC. This effect is recapitulated by treatment with the gut bacterium Roseburia intestinalis (389). Utilizing probiotics, prebiotics, synbiotics, or fecal microbiota transplantation to reverse cancer-promoting dysbiosis represents a key future adjuvant strategy.

Metabolic intervention holds significant promise yet faces substantial challenges: the redundancy and plasticity of metabolic pathways may lead to drug resistance; the systemic side effects of metabolic interventions require stringent monitoring; colorectal cancer with different metabolic subtypes may necessitate personalized combination strategies; and how to optimally sequence or combine various metabolic interventions with immune checkpoint blockade, chemotherapy, and targeted therapy. In the future, leveraging technologies such as single-cell sequencing and spatial metabolomics to deeply map the dynamic landscape of the colorectal cancer immunometabolic microenvironment will guide the development of rational combination therapies. By integrating pharmacological approaches, nutritional strategies, and microbiota modulation to precisely target the metabolic vulnerabilities of CRC from multiple angles, we hold the potential to overcome the current bottlenecks in CRC treatment and offer hope to more patients.

## 6 Discussion and perspectives

Metabolic reprogramming is a defining hallmark of colorectal cancer, driving tumor growth, progression, and immune evasion through the rewiring of glucose, lipid, amino acid, and nucleotide metabolism. This reprogramming not only fulfills the biosynthetic and bioenergetic demands of malignant cells but also actively shapes an immunosuppressive tumor microenvironment. Mechanisms include nutrient competition, accumulation of immunosuppressive metabolites (e.g., lactate, kynurenine), modulation of immune cell function, and intricate crosstalk with gut microbiota, collectively reinforcing malignant traits. These insights provide the foundation for novel metabolic-targeted therapeutic strategies.

### 6.1 Targeting core metabolic pathways represents a primary approach

In glucose metabolism, exploiting the Warburg effect—driven by GLUT1 and glycolytic enzymes like HK2 and PKM2—is pivotal. GLUT1 inhibitors (e.g., BAY-876) reduce glucose uptake, while PKM2 inhibitors (e.g., shikonin) impair energy production and oncogenic signaling. For lipid metabolism, targeting overexpressed enzymes like FASN (inhibitor TVB-3166 limits palmitate synthesis) and SCD1 (inhibitor CAY10566 induces lipotoxicity) shows promise, potentially synergizing with ferroptosis inducers. Cholesterol metabolism offers targets including HMGCR (statins) and SQLE; their inhibition reduces immunosuppressive oxysterols (e.g., 24-OHC), thereby enhancing CD8^+^ T cell activity. Amino acid metabolism interventions include: blocking the glutamine transporter SLC1A5 or glutaminase GLS1 (e.g., V-9302); inhibiting ARG1 or CAT1 to reverse arginine starvation and restore T/NK cell function; and using IDO1/TDO inhibitors to alleviate tryptophan depletion and augment immunotherapy.

### 6.2 Modulating the TME is critical

Strategies include nutrient normalization (e.g., GLUT1 inhibition to increase glucose for CD8^+^ T cells; glutamine supplementation) and reducing harmful metabolites: MCT4 inhibitors (e.g., AZD3965) lower lactate and normalize pH; IDO1 inhibitors and AhR antagonists block kynurenine signaling; SUCNR1 blockers reduce succinate-driven M2 macrophage recruitment; and FH mimetics counter fumarate-induced T cell dysfunction.

### 6.3 Harnessing gut microbiota-metabolism-immunity crosstalk holds significant potential

Harnessing the gut microbiota-metabolism-immunity crosstalk may be achieved through multi-faceted strategies tailored to microbial composition and metabolite profiles. Potential approaches could include: probiotics or fecal microbiota transplantation (FMT) to enrich butyrate-producing Clostridiales, thereby enhancing CD8^+^ T cell activity and immunotherapy responses; precision antibiotics or anti-Fap2 antibodies to target Fusobacterium nucleatum and reduce MDSC infiltration; therapeutic application of beneficial microbial metabolites such as SCFAs and indoles (ILA, IPA); bile acid sequestrants to diminish deleterious secondary bile acids (e.g., DCA); and high-fiber diets or synbiotics to elevate SCFA production, which may complement other therapeutic modalities.

### 6.4 Precision and combination therapies are essential

Biomarker-guided therapeutic selection is crucial for optimizing treatment outcomes. For instance, GLUT1 and GLS1 inhibitors are particularly promising for KRAS-mutant CRC, while FASN inhibitors may be more effective in BRAF-mutant tumors. Combination strategies further enhance therapeutic efficacy: pairing metabolic inhibitors with chemotherapy (such as PKM2 inhibitors combined with 5-FU), immunotherapy (including IDO1 inhibitors used in conjunction with anti-PD-L1 agents), or radiotherapy (for example, SCD1 inhibitors administered alongside ferroptosis inducers) can synergistically amplify anti-tumor effects. Advanced technologies like metabolomics and single-cell sequencing will continue to refine precision medicine approaches by enabling more accurate patient stratification and treatment personalization.

Future advances in single-cell sequencing, spatial transcriptomics, and spatial metabolomics will enable more precise tumor subtyping and refined diagnostic strategies, thereby unlocking greater opportunities for targeted metabolic therapies in colorectal cancer. However, these opportunities are accompanied by significant challenges. First, off-target effects during drug delivery and inherent toxicity to normal tissues necessitate the development of tumor-selective delivery systems with enhanced precision and specificity. Second, tumor cells exhibit remarkable metabolic plasticity—under the pressure of inhibitors, they frequently activate compensatory pathways to evade suppression. Overcoming this barrier requires comprehensive mapping of tumor metabolic networks, which would lay the foundation for co-targeting both primary and compensatory pathways. Third, tumor heterogeneity presents a critical hurdle: distinct immune cell populations within the tumor microenvironment possess divergent metabolic signatures, while tumor cells themselves consist of functionally diverse metabolic subtypes. Therefore, defining the metabolic profiles of these cellular subpopulations and their specific roles in tumorigenesis and progression constitutes a fundamental prerequisite for precision therapeutic intervention.

In summary, metabolic reprogramming offers diverse therapeutic targets for CRC. Strategic targeting of metabolism, TME modulation, leveraging microbiota, and employing precision combinations hold transformative potential. Future research must focus on overcoming current challenges to realize improved patient outcomes.
